# Gradient Matching Methods for Computational Inference in Mechanistic Models for Systems Biology: A Review and Comparative Analysis

**DOI:** 10.3389/fbioe.2015.00180

**Published:** 2015-11-20

**Authors:** Benn Macdonald, Dirk Husmeier

**Affiliations:** ^1^School of Mathematics and Statistics, University of Glasgow, Glasgow, UK

**Keywords:** ordinary differential equations, gradient matching, Gaussian processes, reproducing kernel Hilbert space, parallel tempering, B-splines

## Abstract

Parameter inference in mathematical models of biological pathways, expressed as coupled ordinary differential equations (ODEs), is a challenging problem in contemporary systems biology. Conventional methods involve repeatedly solving the ODEs by numerical integration, which is computationally onerous and does not scale up to complex systems. Aimed at reducing the computational costs, new concepts based on gradient matching have recently been proposed in the computational statistics and machine learning literature. In a preliminary smoothing step, the time series data are interpolated; then, in a second step, the parameters of the ODEs are optimized, so as to minimize some metric measuring the difference between the slopes of the tangents to the interpolants, and the time derivatives from the ODEs. In this way, the ODEs never have to be solved explicitly. This review provides a concise methodological overview of the current state-of-the-art methods for gradient matching in ODEs, followed by an empirical comparative evaluation based on a set of widely used and representative benchmark data.

## 1 Introduction

The elucidation of the structure and dynamics of biopathways is a central objective of systems biology. A standard approach is to view a biopathway as a network of biochemical reactions, which is modeled as a system of ordinary differential equations (ODEs). Following Barenco et al. (2006), this system can typically be expressed as^1^:

(1) $$\frac{dx_{i}\left(t\right)}{\mathrm{dt}}=g_{i}\left(\text{x}\left(t\right),\rho_{i},t\right)-\delta_{i}x_{i}\left(t\right),$$

where *i* ∈ {1, … , *n*} denotes one of *n* components (henceforth referred to as “species”) in the biopathway, *x_i_*(*t*) denotes the concentration of species *i* at time *t*, *δ_i_* is a decay rate and **x**(*t*) is a vector of concentrations of all system components that influence or regulate the concentration of species *i* at time *t*. If, for instance, species *i* is an mRNA, then **x**(*t*) may contain the concentrations of transcription factors (proteins) that bind to the promoter of the gene from which *i* is transcribed. The regulation is modeled by the regulation function *g*. Depending on the species involved, *g* may define different types of regulatory interactions, e.g., mass action kinetics, Michaelis–Menten kinetics, allosteric Hill kinetics, etc. All of these interactions depend on a vector of kinetic parameters, ***ρ****_i_*. For complex biopathways, only a small fraction of ***ρ****_i_* can typically be measured. Hence, the explication of the biopathway dynamics requires the majority of kinetic parameters to be inferred from observed (typically noisy and sparse) time course concentration profiles. In principle, this can be accomplished with standard techniques from machine learning and statistical inference. These techniques are based on first quantifying the difference between predicted and measured time course profiles by some appropriate metric to obtain the likelihood of the data. The parameters are then either optimized to maximize the likelihood (or a regularized version thereof), or sampled from a distribution based on the likelihood (the posterior distribution).

However, the nature of the ODE-based model in equation (1) renders the inference problem computationally challenging in two respects. First, the ODE system often does not permit closed-form solutions. One therefore has to resort to numerical simulations every time the kinetic parameters ***ρ****_i_* are adapted, which is computationally onerous. Second, the likelihood function in the space of parameters ***ρ****_i_* is typically not unimodal, but suffers from multiple local optima. Hence, even if a closed-form solution of the ODEs existed, inference by maximum likelihood would face an NP-hard optimization problem, and Bayesian inference would suffer from poor mixing and convergence of the Markov chain Monte Carlo (MCMC) simulations.

**Figure 1:**
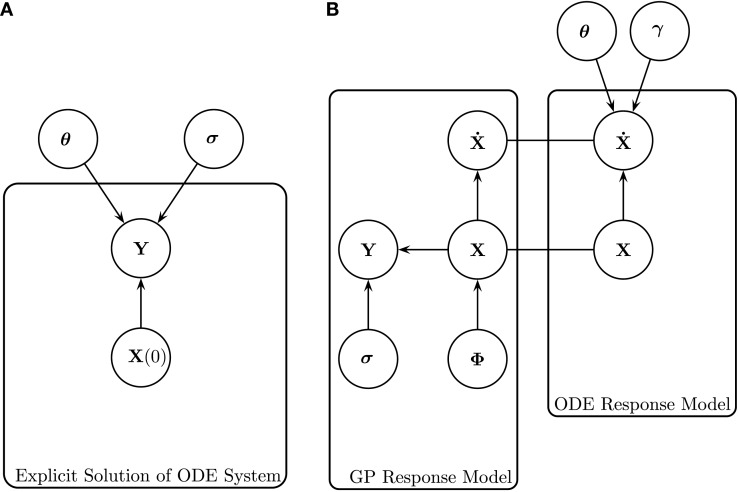
**Graphical representations of (left) the explicit solution of the ODE system, as shown in Calderhead et al. (2008), and (right) gradient matching with Gaussian processes, as proposed in Calderhead et al. (2008) and Dondelinger et al. (2013)**. **(A)** Explicit solution of the ODE system, as shown in Calderhead et al. (2008). The noisy data signals **Y** are described by some initial concentration **X**(0), ODE parameters **θ** and observational errors with SD **σ**. For a given set of initial concentrations **X**(0) and set of ODE parameters **θ**, the ODEs can be integrated to produce a signal, which is then compared to the data signal by some metric defined by the chosen noise model. **(B)** Gradient matching with Gaussian processes, as proposed in Calderhead et al. (2008) and Dondelinger et al. (2013). The gradients $\dot{\text{X}}$ are compared from two modeling approaches; the Gaussian process model and the ODEs themselves. The distribution of **Y** is given in equation (4), the Gaussian process on **X** defined in equation (5), the derivatives of the Gaussian process $\dot{\text{X}}$ in equation (10), the ODE model in equation (2), and the gradient matching in equation (17). All symbols are detailed in Section 2.1.

Conventional inference methods involve numerically integrating the system of ODEs to produce a signal, which is compared to the data by some appropriate metric defined by the chosen noise model, allowing for the calculation of a likelihood. This process is repeated as part of an iterative optimization or sampling procedure to produce estimates of the parameters. Figure 1A is a graphical representation of the model for these conventional inference methods. For a given set of initial concentrations of the entire system **X**(0) and set of ODE parameters ***θ*** [where ***θ*** = (***θ***_1_, … , ***θ**_n_*) and ***θ**_i_* = (***ρ****_i_, δ_i_*)], a signal can be produced by integration of the ODEs. As mentioned previously, for many ODE systems a closed-form solution does not exist, so in practice, numerical integration is implemented instead. Assuming an appropriate noise model (for example, a Gaussian additive noise model) with standard deviation (SD) of the observational error ***σ***, the differences between the resultant signal and the data **Y** can be used to calculate the likelihood of the parameters ***θ***. The process is repeated for different parameters ***θ*** until the maximum likelihood of the parameters is found (in the classical approach) or until convergence to the posterior distribution is reached (in the Bayesian approach). However, the computational costs involved with repeatedly numerically solving the ODEs are large.

To reduce the computational complexity, several authors have adopted an approach based on gradient matching [e.g., Calderhead et al. (2008) and Liang and Wu (2008)]. The idea is based on the following two-step procedure. In a preliminary smoothing step, the time series data are interpolated; then, in a second step, the parameters ***θ*** of the ODEs are optimized so as to minimize some metric measuring the difference between the slopes of the tangents to the interpolants, and the ***θ***-dependent time derivatives from the ODEs. In this way, the ODEs never have to be solved explicitly, and the typically unknown initial conditions are effectively profiled over. A disadvantage of this two-step scheme is that the results of parameter inference critically hinge on the quality of the initial interpolant. A better approach, first suggested in Ramsay et al. (2007), is to regularize the interpolants by the ODEs themselves. Dondelinger et al. (2013) applied this idea to the non-parametric Bayesian approach of Calderhead et al. (2008), using Gaussian processes (GPs), and demonstrated that it substantially improves the accuracy of parameter inference and robustness with respect to noise. As opposed to Ramsay et al. (2007), all smoothness hyperparameters are consistently inferred in the framework of non-parametric Bayesian statistics, dispensing with the need to adopt heuristics and approximations.

This review compares the current state-of-the-art in gradient matching, specifically in the context of parameter inference in ODEs. This comparison aids in understanding the difference between key components of methods without confounding influence from other modeling choices. For instance, we compare the inference paradigm of the parameter that governs the degree of mismatch between the gradients of the interpolants and ODEs [using the method in Dondelinger et al. (2013)] with a tempering approach [from the method in Macdonald and Husmeier (2015)], using the same interpolation scheme (namely, Gaussian processes). This way, we are able to gain an understanding as to what approach may be more suitable, without concern that differences may be due to interpolation choice. If the ODEs provide the correct mathematical description of the system, ideally there should be no difference between the interpolant gradients and those predicted from the ODEs. In practice, however, forcing the gradients to be equal is likely to cause parameter inference techniques to converge to a local optimum of the likelihood. A parallel tempering scheme is the natural way to deal with such local optima, as opposed to inferring the degree of mismatch, since different tempering levels correspond to different strengths of penalizing the mismatch between the gradients. A parallel tempering scheme (which uses smoothed versions of the posterior distribution as well as the usual posterior distribution, see Section 2.2 for more details) was explored by Campbell and Steele (2012).

When comparing one method to another, in order to assess the strengths and weaknesses of an approach, often results are not directly comparable, since different approaches use different methodological paradigms. For example, if the method by Campbell and Steele (2012) (which uses B-splines interpolation) was compared to Dondelinger et al. (2013) (which uses a GP approach) in order to examine the difference between parallel tempering and inference of the parameter controlling the degree of mismatch between the gradients, then the results would be confounded by the choice of interpolation scheme. In this review, we present a comparative evaluation of parallel tempering versus inference in the context of gradient matching for the same modeling framework, i.e., without any confounding influence from the model choice. We also compare the method of Bayesian inference with Gaussian processes with other methodological paradigms, within the specific context of adaptive gradient matching, which is highly relevant to current computational systems biology. We look at the methods of: Campbell and Steele (2012), who carry out parameter inference using adaptive gradient matching and B-splines interpolation; González et al. (2013), who implement a reproducing kernel Hilbert space (RKHS) and penalized maximum likelihood approach in a non-Bayesian fashion; Ramsay et al. (2007), who optimize the gradient mismatch, interpolant, and ODE parameters using a hierarchical regularization method and penalize the difference between the gradients using B-splines in a non-Bayesian approach; Dondelinger et al. (2013), who use adaptive gradient matching with Gaussian processes, inferring the degree of mismatch between the gradients; and Macdonald and Husmeier (2015), who use adaptive gradient matching with Gaussian processes and temper the parameter that controls the degree of mismatch between the gradients.

## 2 Methodology

### 2.1 Adaptive Gradient Matching with Gaussian Processes

The following covers the background of methodology for Dondelinger et al. (2013), and Macdonald and Husmeier (2015), which combines the former method with a parallel tempering scheme for the gradient mismatch parameter (the details on parallel tempering will be given in Section 2.2).

Consider a set of T arbitrary timepoints *t*_1_ < … < *t_T_*, and a set of noisy observations **Y** = (**y**(*t*_1_), … , **y**(*t_T_*)), where **y**(*t*) = **x**(*t*) + **ε**(*t*), n = dim(**x**(*t*)), **X** = (**x**(*t*_1_), … , **x**(*t_T_*)), **y**(*t*) is the data vector of the observations of all species concentrations at time *t*, **x**(*t*) is the vector of the concentrations of all species at time *t*, **y***_i_* is the data vector of the observations of species concentrations *i* at all timepoints, **x***_i_* is the vector of concentrations of species *i* at all timepoints, *y_i_*(*t*) is the observed datapoint of the concentration of species *i* at time *t*, *x_i_*(*t*) is the concentration of species *i* at time *t* and **ε** is multivariate Gaussian noise, $\varepsilon ∼N\left(\text{0},\sigma_{i}^{2}\text{I}\right)$. The signals of the system are described by ordinary differential equations

(2) $${\dot{\text{x}}}_{i}=\frac{d{\text{x}}_{i}}{\mathrm{dt}}=f_{i}\left(\text{X},\theta_{i},\text{t}\right),$$

or alternatively, represented in scalar form

(3) $${\dot{x}}_{i}\left(t\right)=\frac{dx_{i}\left(t\right)}{\mathrm{dt}}=f_{i}\left(\text{x}\left(t\right),\theta_{i},t\right),$$

where ${\dot{\text{x}}}_{i}$ is the vector containing the ODE gradients for species *i* at all timepoints, *f_i_* (**t**) = (*f_i_*(*t*_1_),… ,*f_i_*(*t_T_*))*^Τ^*, ***θ**_i_* = (***ρ****_i_*, *δ_i_*), ***ρ****_i_* is a vector of kinetic parameters, *δ_i_* is a decay rate parameter and *f_i_* (**x**(*t*), ***θ**_i_*, *t*) = *g_i_* (**x**(*t*), ***ρ****_i_*,*t*) −*δ_i_* *x_i_*. Then,

(4) $$p\left(\text{Y}|\text{X},\sigma^{2}\right)=\underset{i}{\prod }\underset{t}{\prod }N\left(y_{i}\left(t\right)|x_{i}\left(t\right),\sigma_{i}^{2}\right),$$

and the matrices **X** and **Y** are of dimension n by T. Following Calderhead et al. (2008), we place a Gaussian process (GP) prior on **x***_i_*,

(5) $$p\left({\text{x}}_{i}|\mu_{i},\phi_{i}\right)=N\left({\text{x}}_{i}|\mu_{i},{\text{C}}_{\phi_{i}}\right),$$

where ***μ****_i_* is a mean vector, for simplicity set as the sample mean, and ${\text{C}}_{\phi_{i}}$ is a positive definite matrix of covariance functions with hyperparameters *ϕ_i_*. Since differentiation is a linear operation, a Gaussian process is closed under differentiation, and the joint distribution of the state variables **x***_i_* and their time derivatives $\dot{\text{x}}$*_i_* is multivariate Gaussian with mean vector (***μ**_i_*, **0**)*^Τ^* and covariance functions

(6) $$\mathrm{cov}\left[x_{i}\left(t\right),x_{i}\left(t\prime \right)\right]=C_{\phi_{i}}\left(t,t\prime \right),$$

(7) $$\mathrm{cov}\left[{\dot{x}}_{i}\left(t\right),x_{i}\left(t\prime \right)\right]=\frac{\partial C_{\phi_{i}}\left(t,t\prime \right)}{\mathrm{\partial t}}\;:=\;C_{\phi_{i}}^{\prime }\left(t,t\prime \right),$$

(8) $$\mathrm{cov}\left[x_{i}\left(t\right),{\dot{x}}_{i}\left(t\prime \right)\right]=\frac{\partial C_{\phi_{i}}\left(t,t\prime \right)}{\mathrm{\partial t}\prime }\;:=\;\prime C_{\phi_{i}}\left(t,t\prime \right),$$

(9) $$\mathrm{cov}\left[{\dot{x}}_{i}\left(t\right),{\dot{x}}_{i}\left(t\prime \right)\right]=\frac{\partial^{2}C_{\phi_{i}}\left(t,t\prime \right)}{\mathrm{\partial t\partial t}\prime }\;:=\;C_{\phi_{i}}^{\prime \prime }\left(t,t\prime \right),$$

where $C_{\phi_{i}}\left(t,t^{\prime }\right)$ are the elements of the covariance matrix ${\text{C}}_{\phi_{i}}$. Using elementary transformations of Gaussian distributions [for example, see page 87 of Bishop (2006)], the conditional distribution for the state derivatives is then

(10) $$p\left({\dot{\text{x}}}_{i}|{\text{x}}_{i},\mu_{i},\phi_{i}\right)=N\left({\text{m}}_{i},{\text{K}}_{i}\right),$$

where

(11) $${\text{m}}_{i}={\;}^{\prime }{\text{C}}_{\phi_{i}}{\text{C}}_{\phi_{i}}{\;}^{-1}\left({\text{x}}_{i}-\mu_{i}\right)\;\text{and}\;{\text{K}}_{i}={\text{C}}_{\phi_{i}}^{\prime \prime }-{\;}^{\prime }{\text{C}}_{\phi_{i}}{\text{C}}_{\phi_{i}}{\;}^{-1}{\text{C}}_{\phi_{i}}^{\prime }.$$

Assuming additive Gaussian noise with a state-specific error variance *γ_i_*, from equation (2) we get

(12) $$p\left({\dot{\text{x}}}_{i}|\text{X},\theta_{i},\gamma_{i}\right)=N\left(f_{i}\left(\text{X},\theta_{i},\text{t}\right),\gamma_{i}\text{I}\right).$$

Calderhead et al. (2008), and Dondelinger et al. (2013) link the interpolant in equation (10) with the ODE model in equation (12) using a product of experts approach, as illustrated in Figure 1B, obtaining the following distribution

(13) $$p\left({\dot{\text{x}}}_{i}|\text{X},\theta_{i},\mu_{i},\phi_{i},\gamma_{i}\right)\propto p\left({\dot{\text{x}}}_{i}|{\text{x}}_{i},\mu_{i},\phi_{i}\right)p\left({\dot{\text{x}}}_{i}|\text{X},\theta_{i},\gamma_{i}\right)=N\left({\text{m}}_{i},{\text{K}}_{i}\right)N\left(f_{i}\left(\text{X},\theta_{i},\text{t}\right),\gamma_{i}\text{I}\right).$$

The joint distribution is therefore

(14) $$p\left(\dot{\text{X}},\text{X},\theta ,\mu ,\phi ,\gamma \right)=p\left(\theta \right)p\left(\phi \right)p\left(\gamma \right)\underset{i}{\prod }p\left({\dot{\text{x}}}_{i}|\text{X},\theta_{i},\mu_{i},\phi_{i},\gamma_{i}\right)p\left({\text{x}}_{i}|\phi_{i}\right),$$

where *γ* is the vector containing all the gradient mismatch parameters and p(***θ***), *p*(***ϕ***), *p*(*γ*) are the priors over the respective parameters. Dondelinger et al. (2013) show that you can marginalize over the derivatives to get a closed-form solution to

(15) $$p\left(\text{X},\theta ,\mu ,\phi ,\gamma \right)=\int p\left(\dot{\text{X}},\text{X},\theta ,\mu ,\phi ,\gamma \right)d\dot{\text{X}}.$$

Using equations (4) and (15), our full joint distribution becomes

(16) $$p\left(\text{Y},\text{X},\theta ,\mu ,\phi ,\gamma ,\sigma^{2}\right)=p\left(\text{Y}|\text{X},\sigma^{2}\right)p\left(\text{X}|\theta ,\mu ,\phi ,\gamma \right)p\left(\theta \right)p\left(\phi \right)p\left(\gamma \right)p\left(\sigma^{2}\right),$$

where the likelihood *p*(**Y**|**X**, ***σ***) is defined in equation (4) and *p*(***σ***^2^) is the prior over the variances of the observational error. Dondelinger et al. (2013) show

(17) $$p\left(\text{X}|\theta ,\mu ,\phi ,\gamma \right)\propto \frac{1}{Z}\exp\left[\;-\frac{1}{2}\underset{i}{\sum }\;\;\left({\text{x}}_{i}^{T}{\text{C}}_{\phi_{i}}{\text{x}}_{i}+{\left({\text{f}}_{i}-{\text{m}}_{i}\right)}^{T}{\left({\text{K}}_{i}+\gamma_{i}\text{I}\right)}^{-1}\left({\text{f}}_{i}-{\text{m}}_{i}\right)\;\right)\;\;\right]\;,$$

where $Z=\prod_{i}{\left|2\pi \left({\text{K}}_{i}+\gamma_{i}\text{I}\right)\right|}^{\frac{1}{2}}$ and **f***_i_* is the vector containing the gradients from the ODEs for species *i*. The sampling is conducted using MCMC, where the whitening approach of Murray and Adams (2010) is used to efficiently sample in the joint space of GP hyperparameters ***ϕ*** and latent variables **X**. The concept of gradient matching with Gaussian processes can be seen graphically in Figure 1B. The data **Y** are explained by the latent variables **X**, which are modeled by a Gaussian process with hyperparameters ***ϕ***, and SD of the observational errors ***σ***. The gradients from the ODE model are compared to those from the Gaussian process, subject to some degree of mismatch controlled by parameter **γ**, dispensing with the need to explicitly solve the ODEs.

### 2.2 Parallel Tempering

A challenging problem, which sampling methods face, is that of local optima. The aim of sampling is to represent fully the configuration space weighted by the volume of the corresponding posterior density peaks. In order to do this, the sampling algorithm implemented must be able to adequately explore the posterior distribution. If this landscape is rugged, with many local optima and low-probability barriers separating areas of high posterior probability, mixing and convergence of the Markov chain Monte Carlo simulations can be poor. For example, consider the Metropolis–Hastings algorithm, which proposes a move and computes the acceptance probability *p*_move_ by taking the ratio of the posterior densities of the proposed state to the current state. If *p*_move_* * > 1, the algorithm accepts the proposed move. If *p*_move_* * < 1, the proposed state is accepted with probability *p*_move_. If then, the parameter location of the algorithm is currently situated at a local optimum, then the proposed move could result in a small *p*_move_. Theoretically, the algorithm will eventually be able to move the parameter location out of this region; however, in practice, this could take a considerable amount of time. If the total number of MCMC iterations has been specified in advance, the simulation could finish before the parameter position of the algorithm has escaped the local optimum and explored the remainder of the region. Entrapment in local optima can mislead established convergence tests and erroneously indicate a sufficient degree of convergence.

**Figure 2:**
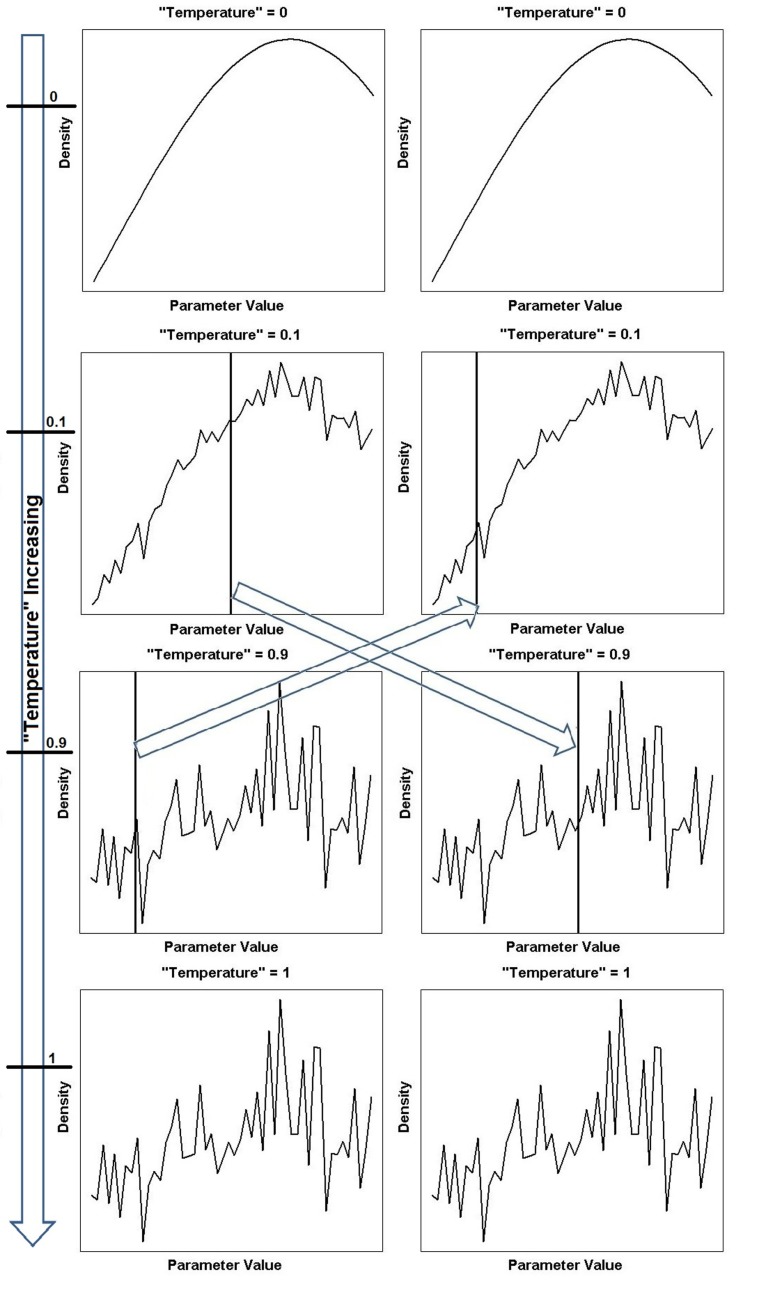
**Continued A one-dimensional illustration of equation (18) showing different power posterior distributions for different levels or “temperatures” of the likelihood**. The posterior landscape is smoother at lower “temperatures” (corresponding to chains closer to the prior) and becomes increasingly rugged until the true posterior landscape is recovered for “temperature” = 1. The arrow on the far left depicts the increase in “temperature” and the horizontal ticks mark the specific “temperature” of that chain. Two chains (“temperature” = 0.1 and “temperature” = 0.9) have been chosen to swap parameter locations (locations indicated by vertical line). The left column shows the parameter locations of the tempering algorithm before the swap and the right column shows the parameter locations of the tempering algorithm after the swap. The swapping of locations is indicated by the arrows in the center of the figure.

Parallel tempering is a method that tackles the problem of local optima. It involves running multiple MCMC simulations at different levels or “temperatures”^2^ of the likelihood in parallel. Low “temperatures” flatten the posterior landscape, making it easier to explore the region, since the peaks have been smoothed. This can be seen graphically in Figure 2. As the “temperature” is increased to the highest value, the landscape becomes more rugged and eventually the original posterior landscape is recovered (see bottom of Figure 2).

At every MCMC iteration, two “temperature” chains are chosen and the parameter locations where the sampling algorithm is currently situated are swapped, see middle of Figure 2. This way, the algorithm can move the parameter position from a local optimum to somewhere else on the posterior landscape, dispensing with the need to gradually navigate away from the region and the problems associated with doing so.

Consider a series of “temperatures”, 0 = *β*^(1)^ < … < *β*^(^*^M^*^)^ = 1 and a power posterior distribution of our ODE parameters [Friel and Pettitt (2008)]

(18) $$p_{\beta^{\left(j\right)}}\left(\theta^{\left(j\right)}|\text{y}\right)\propto p\left(\theta^{\left(j\right)}\right)p{\left(\text{y}|\theta^{\left(j\right)}\right)}^{\beta^{\left(j\right)}}.$$

Equation (18) reduces to the prior for *β*^(^*^j^*^)^ = 0 (see top of Figure 2), and becomes the posterior when *β*^(^*^j^*^)^ = 1 (see bottom of Figure 2), with 0 < *β*^(^*^j^*^)^ < 1 creating a distribution between our prior and posterior (see Figure 2). The M *β*^(^*^j^*^)^ annealed likelihoods in equation (18) are used as the target densities of M parallel MCMC chains [Campbell and Steele (2012)]. At each MCMC step, each “temperature” chain independently performs a Metropolis–Hastings step to update ***θ***^(^*^j^*^)^, the parameter vector associated with temperature *β*^(^*^j^*^)^

(19) $$p_{\text{move}}=\text{min}\left(1,\frac{\begin{array}{c} p{\left(\text{y}|\theta^{\text{proposed}\left(j\right)}\right)}^{\beta^{\left(j\right)}}p\left(\theta^{\text{proposed}\left(j\right)}\right)\times q\left(\theta^{\text{current}\left(\text{j}\right)}|\theta^{\text{proposed}\left(\text{j}\right)}\right) \end{array}}{\begin{array}{c} p{\left(\text{y}|\theta^{\text{current}\left(j\right)}\right)}^{\beta^{\left(j\right)}}p\left(\theta^{\text{current}\left(j\right)}\right)\times q\left(\theta^{\text{proposed}\left(\text{j}\right)}|\theta^{\text{current}\left(\text{j}\right)}\right) \end{array}}\right),$$

where *q*( ) is the proposal distribution and the superscripts “proposed” and “current” indicate whether the algorithm is being evaluated at the proposed or current state. Also, at each MCMC step, two chains are randomly selected, and a proposal to exchange parameters is made, with acceptance probability

(20) $$p_{\text{swap}}=\text{min}\left(1,\frac{p_{\beta^{\left(k\right)}}\left(\theta^{\left(j\right)}|\text{y}\right)p_{\beta^{\left(j\right)}}\left(\theta^{\left(k\right)}|\text{y}\right)}{p_{\beta^{\left(j\right)}}\left(\theta^{\left(j\right)}|\text{y}\right)p_{\beta^{\left(k\right)}}\left(\theta^{\left(k\right)}|\text{y}\right)}\right).$$

A graphical representation of the swap moves between chains can be seen in Figure 2.

The method by Macdonald and Husmeier (2015) focuses on the intrinsic slack parameter *γ_i_* [see equation (12)], which theoretically should be *γ_i_* = 0, since this corresponds to no mismatch between the gradients. In practice, it is allowed to take on larger values, *γ_i_* > 0, to prevent the inference scheme from getting stuck in sub-optimal states. However, rather than inferring *γ_i_* like a model parameter, as carried out in Dondelinger et al. (2013), other authors [e.g., Campbell and Steele (2012)] propose that *γ_i_* should be gradually set to zero, since values closer to zero force the gradients to be more similar and tie the interpolants closer to the ODEs. It is possible to abruptly set the values to zero, rather than gradually; however, this is likely to cause the parameter inference techniques to converge to a local optimum of the likelihood. To this end, Macdonald and Husmeier (2015) combine the gradient matching with Gaussian processes approach in Dondelinger et al. (2013) with the tempering approach in Campbell and Steele (2012) and temper this parameter to zero.

We choose values of *γ_i_* and assign them to the variance parameter in equation (12) for each “temperature” *β*^(^*^j^*^)^, such that chains closer to the prior (*β*^(^*^j^*^)^ closer to 0) allow the gradients from the interpolant to have more freedom to deviate from those predicted by the ODEs (which corresponds to a larger *γ_i_*), chains closer to the posterior (*β*^(^*^j^*^)^ closer to 1) more closely match the gradients (corresponding to a smaller *γ_i_*), and for the chain corresponding to *β*^(^*^M^*^)^ = 1, we wish that the mismatch is approximately zero (*γ_i_* ≈ 0). Since *γ_i_* corresponds to the variance of our state-specific error [see equation (12)], as *γ_i_* → 0, we have less mismatch between the gradients, and as *γ_i_* gets larger, the gradients have more freedom to deviate from one another. Hence, we temper *γ_i_* toward zero. Now, each *β*^(^*^j^*^)^ chain in equation (18) has a γ_i_^(j)^ [where the superscript (*j*) indicates the gradient mismatch parameter associated with “temperature” *β*^(^*^j^*^)^] fixed in place for the strength of the gradient mismatch.

Continuing the notation, anything with a superscript (*j*) is the associated variable or fixed parameter for “temperature” chain *β*^(^*^j^*^)^. The ODE model in equation (12) now becomes

(21) $$p\left({\dot{\text{x}}}_{i}^{\left(j\right)}|{\text{X}}^{\left(j\right)},\theta_{i}^{\left(j\right)},\gamma_{i}^{\left(j\right)}\right)=N\left(f_{i}^{\left(j\right)}\left({\text{X}}^{\left(j\right)},\theta_{i}^{\left(j\right)},\text{t}\right),\gamma_{i}^{\left(j\right)}\text{I}\right),$$

where this distribution is evaluated at each of the *j* chains. Following equations (13)–(16), we obtain for the joint distribution

(22) $$\begin{array}{l} p\left(\text{Y},{\text{X}}^{\left(j\right)},\theta^{\left(j\right)},\mu ,\phi^{\left(j\right)},\gamma^{\left(j\right)},\sigma^{2\left(j\right)}\right)=p{\left(\text{Y}|{\text{X}}^{\left(j\right)},\sigma^{2\left(j\right)}\right)}^{\beta^{\left(j\right)}}\\ \;\times p\left({\text{X}}^{\left(j\right)}|\theta^{\left(j\right)},\mu ,\phi^{\left(j\right)},\gamma^{\left(j\right)}\right)p\left(\theta^{\left(j\right)}\right)p\left(\phi^{\left(j\right)}\right)p\left(\sigma^{2\left(j\right)}\right). \end{array}$$

Equation (22) is calculated for each of the *j* chains. The particular schedules used for *γ_i_* in this review are given in Table 1. For more details on tempering, see Calderhead and Girolami (2009) and Mohamed et al. (2012). The computational times for the methods from Dondelinger et al. (2013) and Macdonald and Husmeier (2015), in comparison to numerically integrating the ODEs, can be found in Table 2.

**Table 1:** **Ranges of the penalty parameter **γ**_*i*_ for LB2 and LB10**.

Method	Chains	Range of penalty γ	Method	Chains	Range of penalty γ
LB2	4	[1, 0.125]	LB10	4	[1, 0.001]
LB2	10	[1, 0.00195]	LB10	10	[1, 1 × 10^−9^]

*In this review, **γ**_i_ = **γ**∀i*.

**Table 2:** **Computational times for INF and a method that numerically integrates the ODEs for the protein signaling transduction pathway in equations (63)–(67)**.

INF		Numerical integration	

Exectution time of 1 **×** 10^5^ MCMC steps (seconds)		Execution time of 1 **×** 10^5^ MCMC steps (seconds)	
Median	Interquartile range	Median	Interquartile range
2500	[2400, 2600]	12,500	[12,000, 13,000]

**Number of steps until convergence**		**Number of steps until convergence**	
**Median**	**Interquartile range**	**Median**	**Interquartile range**

3.5 × 10^4^	[3.25 × 10^4^, 4.5 × 10^4^]	7.9 × 10^4^	[7.5 × 10^4^, 8.25 × 10^4^]

*Table constructed from the boxplots in Dondelinger et al. (2013). The LB2 and LB10 methods were equivalent to INF in terms of computational time*.

### 2.3 B-Splines

Splines are used for function interpolation, where the function of interest is approximated by a weighted linear combination of basis functions. These basis functions, called “splines”, are “local” polynomials, where the exact functional form depends on the particular type of spline that is used (for example, a truncated power basis). See Hastie et al. (2009) for an overview of different types of splines.

The advantage of spline interpolation over global polynomial interpolation is that the interpolation error can be made small even when using low degree polynomials for the splines. This, in particular, avoids the problem of Runge’s phenomenon, in which oscillations can occur between data points when interpolating using high degree polynomials.

B-splines interpolation takes the form

(23) $$x\left(t\right)=\overset{m}{\underset{i=0}{\sum }}\alpha_{i}\phi_{i,d}\left(t\right),$$

where *m* + 1 is the number of basis functions, *d* is the degree of polynomial, *α_i_* is a coefficient and *ϕ_i,d_* (*t*) is the *i*^th^ basis function of polynomial degree *d* evaluated at time *t*. For some vector of fixed points called knots [denoted ***τ***, where *x*(*t*) is continuous at each knot], the basis functions are calculated with the following recursive formulae

(24) $$\phi_{i},0\left(t\right)=\left\{ \begin{array}{l} 1\;\text{if}\;\tau_{i}\leq t<\tau_{i}+1\\ 0\;\text{otherwise} \end{array}\right.$$

(25) $$\phi_{i,d}\left(t\right)=\frac{t-\tau_{i}}{\tau_{i+d}-\tau_{i}}\phi_{i,d-1}\left(t\right)+\frac{\tau_{i+d+1}-t}{\tau_{i+d+1}-\tau_{i+1}}\phi_{i+1,d-1}\left(t\right).$$

The coefficients *α_i_* are then estimated by

(26) $$\hat{\alpha }={\left(\Phi^{T}\Phi \right)}^{-1}\Phi^{T}\text{y},$$

where $\hat{\alpha }$ is the vector containing all the coefficients (and *α_i_* would correspond to the (*i *+ 1)^th^ position in the vector) and **Φ** is the matrix containing all the basis functions

(27) $$\Phi =\left[\begin{array}{ccc} \phi_{0,d}\left(t_{1}\right) & \ldots & \phi_{m,d}\left(t_{1}\right)\\ \vdots & \ddots & \vdots \\ \phi_{0,d}\left(t_{T}\right) & \ldots & \phi_{m,d}\left(t_{T}\right) \end{array}\right].$$

One can aim to avoid over-fitting by penalizing the 2nd derivative of the function *x*(*t*) (known as penalized splines), making our objective function

(28) $$J\left(x\right)=\overset{N}{\underset{s=1}{\sum }}{\left(y\left(t_{s}\right)-x\left(t_{s}\right)\right)}^{2}+\lambda \int {\left(\frac{d^{2}x}{dt^{2}}\right)}^{2}\mathrm{dt},$$

where *λ* controls the amount of trade-off between the data fit and penalty term. In this case, the coefficients *α_i_* are estimated by

(29) $$\hat{\alpha }={\left(\Phi^{T}\Phi +\lambda \text{D}\right)}^{-1}\Phi^{T}\text{y},$$

where **D** is the solution to the penalty in equation (28) (the integral of the square of the second derivative of *x*). It is possible to change the penalty term in equation (28) to some other penalty form (this is known as P-splines), where the **D** in equation (29) would be updated accordingly.

### 2.4 Smooth Functional Tempering

Here, we detail the method for parameter inference used in Campbell and Steele (2012). In their paper, the authors discuss two types of smooth functional tempering, one that needs to infer the initial conditions of the species concentrations and one that does not. This review uses the method that does not need to infer the initial conditions. If the initial conditions are unknown, then they must be inferred as an extra parameter in the inference procedure; however, the method described in this section effectively profiles over the initial conditions, dispensing with the need to infer them. This reduces the complexity of the procedure, which is more appealing. The reader can refer to the original publication should they wish to implement the former procedure. The choice of interpolation scheme for the concentrations **x***_i_* is B-splines. For an introduction to parallel tempering, see Section 2.2.

The posterior distribution of the parameters is

(30) $$p_{\beta^{\left(j\right)}}\left(\theta^{\left(j\right)},\sigma^{2\left(j\right)}|\text{Y},{\text{X}}^{\left(j\right)}\right)\propto p\left(\theta^{\left(j\right)},\sigma^{2\left(j\right)}\right)p\left({\text{X}}^{\left(j\right)}|\theta^{\left(j\right)},\lambda^{\left(j\right)}\right)p{\left(\text{Y}|{\text{X}}^{\left(j\right)},\sigma^{2\left(j\right)}\right)}^{\beta^{\left(j\right)}},$$

where the superscript *j* denotes those variables associated with “temperature” *β*^(^*^j^*^)^, the likelihood, *p*(**Y**|**X**^(^*^j^*^)^, **σ**^2(^*^j^*^)^) = *N*(**X**^(^*^j^*^)^, **σ**^2(^*^j^*^)^), is tempered in the same way as in equation (18), ***λ*** = (*λ*_1_, … , *λ_n_*) and *p*(**X**^(^*^j^*^)^|***θ***^(^*^j^*^)^, ***λ***^(^*^j^*^)^) is

(31) $$p\left({\text{X}}^{\left(j\right)}|\theta^{\left(j\right)},\lambda^{\left(j\right)}\right)=\exp\left[-\overset{n}{\underset{i=1}{\sum }}\lambda_{i}^{\left(j\right)}||{\overset{˙}{\text{x}}}_{i}^{\left(j\right)}-f_{i}^{\left(j\right)}\left({\text{X}}^{\left(j\right)},\theta_{i}^{\left(j\right)},\text{t}\right)|{|}^{2}\right],$$

which is analogous to

(32) $$p\left({\text{X}}^{\left(j\right)}|\theta^{\left(j\right)},\lambda^{\left(j\right)}\right)=\exp\left[-\overset{n}{\underset{i=1}{\sum }}\lambda_{i}^{\left(j\right)}\overset{T}{\underset{t=1}{\sum }}{\left({\dot{x}}_{i}^{\left(j\right)}\left(t\right)-f_{i}^{\left(j\right)}\left({\text{x}}^{\left(j\right)}\left(t\right),\theta_{i}^{\left(j\right)},t\right)\right)}^{2}\right].$$

For details on tempering, see Section 2.2. In equation (31), ${\lambda_{i}}^{\left(j\right)}$ is the gradient mismatch parameter for species *i* corresponding to “temperature” *β*^(^*^j^*^)^ (similar to the mismatch parameter ${\gamma_{i}}^{\left(j\right)}$ in Section 2.1). The ${\lambda_{i}}^{\left(j\right)}$ is chosen in advance and fixed to each “temperature” *β*^(^*^j^*^)^ such that $0<{\lambda_{i}}^{\left(1\right)}\leq \cdots \leq {\lambda_{i}}^{\left(M\right)}\leq \infty$, where values closer to 0 allow the gradients to be more different to one another and values closer to ∞ restrict them from being different.

Sampling from equation (30) is performed using MCMC.

### 2.5 Penalized Likelihood with Hierarchical Regularization

**Figure 3:**
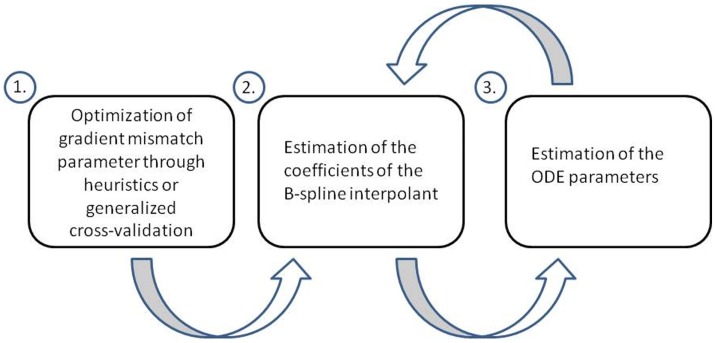
**Flow chart of the three level approach employed by Ramsay et al. (2007)**. At level 1, the gradient mismatch parameter is optimized either by visual or numerical heuristics or through generalized cross-validation. At level 2, the coefficients of the interpolant are estimated (splines in this method). At level 3, the ODE parameters are estimated. Levels 2 and 3 are iterated using a pseudo-delta method (see Section 2.5 for details).

Ramsay et al. (2007) aim to conduct parameter inference in ODEs using a penalized likelihood approach and a hierarchical regularization in order to tune the gradient mismatch parameter and parameters of their interpolation scheme (splines). They perform parameter inference in a hierarchical three level approach. At level 1, they optimize the gradient mismatch parameter, in order to ensure the estimates of the coefficients of their interpolant are properly regularized by the mismatch to the ODEs. In their paper, they adjust the gradient mismatch parameter manually using numerical and visual heuristics, but suggest a way it could be achieved through generalized cross-validation, which we will detail. At level 2, the coefficients of the interpolant are optimized. While optimizing for the parameters in the final step, each time the ODE parameters and observational noise parameters are changed, they re-optimize the coefficients of the interpolant, by penalizing the differences between the gradients, which allows the ODEs to regulate the interpolant. At level 3, the ODE and observational noise parameters are estimated using a sum of squares criterion. This criterion is optimized directly for the ODE and observational noise parameters, but it is also optimized implicitly, since the sum of squares incorporates **x***_i_*, which itself was optimized with respect to these parameters at level 2. A flow chart of these three levels can be found in Figure 3.

At level 1 of the three hierarchical levels, the gradient mismatch parameter is configured. To avoid the need for heuristics, Ramsay et al. (2007) suggest the use of generalized cross-validation, since the estimation of the state variables for some gradient mismatch parameter *λ* is usually a non-linear problem and so standard cross-validation methods are not applicable. Generalized cross-validation takes the form

(33) $$F\left(\lambda \right)=\frac{\sum_{i=1}^{n}||{\text{y}}_{i}-{\text{x}}_{i}|{|}^{2}}{{\left[\sum_{i=1}^{n}\left\{T-\sum_{t=1}^{T}\frac{dx_{i}\left(t\right)}{dy_{i}\left(t\right)}\right\}\right]}^{2}},$$

where **y***_i_* is the data for species *i*, **x***_i_* is the interpolant corresponding to species *i*, *n* is the number of species and *T* is the number of timepoints. The derivatives in the denominator can be expressed as

(34) $$\frac{dx_{i}\left(t\right)}{dy_{i}\left(t\right)}=\frac{\partial x_{i}\left(t\right)}{\partial \alpha }\frac{d\alpha }{dy_{i}\left(t\right)},$$

where ***α*** are the estimated coefficients of the splines interpolant [see equation (29)]. Calculating these derivatives takes the dependency of the data **y** and the ODE parameters ***θ*** into account, since $\frac{d\alpha }{d\text{y}}=\frac{\partial \alpha }{\partial \theta }\frac{d\theta }{d\text{y}}+\frac{\partial \alpha }{\partial \text{y}}$. The estimates of ***λ*** will be calculated by minimizing equation (33) over values of ***λ***.

The second level involves estimating the coefficients of the splines interpolant using the following criterion

(35) $$J\left(\alpha |\theta ,\sigma ,\lambda \right)=\overset{n}{\underset{i=1}{\sum }}w_{i}||{\text{y}}_{i}-{\text{x}}_{i}|{|}^{2}+\overset{n}{\underset{i=1}{\sum }}\lambda_{i}\int {\left[\frac{dx_{i}\left(t\right)}{\mathrm{dt}}-f_{i}\left(\text{x}\left(t\right),\theta_{i},t\right)\right]}^{2}\mathrm{dt},$$

where $\frac{d{\text{x}}_{i}}{\mathrm{dt}}$ is the gradient of the interpolant for species *i* and *w_i_* are weights to normalize the sum of squares of different species (so that species on varying scales of measurement do not distort the sum of squares with very large or very small residuals that are simply a consequence of their magnitude or unit of measurement). Large values of *λ_i_* mean that the gradients have to more closely match one another (since the difference between them will need to tend to 0, to compensate for the large penalty a large *λ_i_* would produce), whereas small values would allow the gradients to differ more. The penalty term in equation (35) allows the mismatch between the gradients to regularize the estimates of the interpolant coefficients.

At the third level, the ODE parameters are optimized using the sum of squares criterion

(36) $$S\left(\theta |\lambda \right)=\overset{n}{\underset{i=1}{\sum }}w_{i}||{\text{y}}_{i}-{\text{x}}_{i}|{|}^{2}.$$

To optimize equation (36) with respect to ***θ***, Ramsay et al. (2007) find the solution of the gradient

(37) $$\frac{\mathrm{dS}\left(\theta |\lambda \right)}{d\theta }=\frac{\mathrm{\partial S}\left(\theta |\lambda \right)}{\partial \theta }+\frac{\mathrm{\partial S}\left(\theta |\lambda \right)}{\partial \alpha }\frac{d\alpha }{d\theta }=0.$$

Since the function ***α***(***θ***) is not explicitly available, $\frac{d\alpha }{d\theta }$ is calculated by application of the implicit function theorem of differential calculus. This gives

(38) $$\frac{d\alpha }{d\theta }=-{\left(\frac{\partial^{2}J\left(\alpha |\theta ,\sigma ,\lambda \right)}{\partial \alpha^{2}}\right)}^{-1}\frac{\partial^{2}J\left(\alpha |\theta ,\sigma ,\lambda \right)}{\partial \alpha \partial \theta }.$$

### 2.6 Reproducing Kernel Hilbert Space

Here, we provide background for reproducing kernel Hilbert spaces (RKHS) that are used in González et al. (2013), and how they compare to Gaussian processes. RKHS interpolation is a useful tool in statistical learning, since a property of reproducing kernel Hilbert spaces, known as the representer theorem (details to follow), means that every function in an RKHS can be written as a linear combination of the kernel function evaluated at the training points. This provides a computationally fast process for interpolation, which is particularly useful in gradient matching, since the original purpose of gradient matching is to obtain a computational speed-up over methods involving calculating numerical solutions to the ODEs.

By Mercer’s theorem [Mercer (1909)], we are able to represent a kernel that produces a positive definite covariance matrix in terms of eigenvalues *λ_s_* and eigenfunctions *v_s_*

(39) $$k\left(t_{i},t_{j}\right)=\overset{\infty }{\underset{s=1}{\sum }}\lambda_{s}\nu_{s}\left(t_{i}\right)\nu_{s}\left(t_{j}\right).$$

These *v_s_* form an orthonormal basis for a function space

(40) $$H=\left\{f:f\left(t\right)=\overset{\infty }{\underset{s=1}{\sum }}f_{s}\nu_{s}\left(t\right),\overset{\infty }{\underset{s=1}{\sum }}\frac{f_{s}^{2}}{\lambda_{s}}<\infty \right\}.$$

The inner product between two functions $f\left(t\right)=\sum_{s=1}^{\infty }f_{s}\nu_{s}\left(t\right)$ and $g\left(t\right)=\sum_{s=1}^{\infty }g_{s}\nu_{s}\left(t\right)$ in the space in equation (40) is defined as

(41) $${\langle f,g\rangle }_{H}\;≜\overset{\infty }{\underset{s=1}{\sum }}\frac{f_{s}g_{s}}{\lambda_{s}},$$

which Murphy (2012) shows implies that

(42) $$\langle k\left(t_{1},\cdot \right),k\left(t_{2},\cdot \right)\rangle_{H}=k\left(t_{1},t_{2}\right).$$

This is known as the reproducing property and the space of functions *H* is called a reproducing kernel Hilbert space. Now consider the minimization problem

(43) $$J\left(f\right)=\frac{1}{2\sigma^{2}}\overset{N}{\underset{s=1}{\sum }}{\left(y_{s}-f\left(t_{s}\right)\right)}^{2}+\frac{1}{2}||f|{|}_{H}^{2},$$

where *J*(*f*) is the objective function and ||*f* ||*_H_* is the norm in Hilbert space

(44) $$||f|{|}_{H}={\langle f,f\rangle }_{H}=\overset{\infty }{\underset{s=1}{\sum }}\frac{f_{s}^{2}}{\lambda_{s}}.$$

The desired function used for interpolation should be simple and provide a good fit to the data. Complex functions with respect to the kernel in equation (39) will produce large norms, since they will need many eigenfunctions to represent them, and therefore be more heavily penalized in equation (43). Schöelkopf and Smola (2002) show that the desired function must have the following form

(45) $$f\left(t\right)=\overset{N}{\underset{s=1}{\sum }}c_{s}k\left(t,t_{s}\right).$$

This is known as the representer theorem. To solve for **c**, we combine equation (45) with equation (43), giving us

(46) $$J\left(\text{c}\right)=\frac{1}{2\sigma^{2}}|\text{y}-\text{Kc}{|}^{2}+\frac{1}{2}{\text{c}}^{T}\text{Kc},$$

where **K** is a matrix of kernel elements for all combinations of observed timepoints. Minimizing with respect to **c** gives us

(47) $$\hat{\text{c}}={\left(\text{K}+\sigma^{2}\text{I}\right)}^{-1}\text{y}.$$

Hence,

(48) $$\hat{f}\left(t_{*}\right)=\overset{N}{\underset{s=1}{\sum }}{\hat{c}}_{s}k\left(t_{*},t_{s}\right)={\text{k}}_{*}^{\text{T}}{\left(\text{K}+\sigma^{2}\text{I}\right)}^{-1}\text{y},$$

where t_*_ is the timepoint at which one wants to make predictions and k_*_ is the vector of kernel elements for all combinations of t_*_ and *t_s_*. This form is the same as a posterior mean of a Gaussian process predictive distribution.

### 2.7 Penalized Likelihood with RKHS

The goal of González et al. (2013) is to create a penalized likelihood function that incorporates the information of the ODEs, then using the properties of reproducing kernel Hilbert spaces to perform parameter estimation in a computationally fast manner. They consider ODEs of the form

(49) $${\dot{\text{x}}}_{i}=g_{i}\left(\text{Z},\rho_{i},\text{t}\right)-\delta_{i}{\text{x}}_{i},$$

or alternatively, represented in scalar form

(50) $${\dot{x}}_{i}\left(t\right)=g_{i}\left(\text{z}\left(t\right),\rho_{i},t\right)-\delta_{i}x_{i}\left(t\right),$$

where **x***_i_* is the vector of mRNA concentrations for species *i*, *δ_i_* is the degradation rate of the mRNA concentrations for species *i*, **Z** is the matrix containing the concentrations of all proteins [transcription factors (TFs)] at all timepoints, **z**(*t*) is the vector containing the concentrations of all proteins at timepoint *t*, ***ρ****_i_* is a parameter vector that governs the amount of regulation that the TFs have on the *i*^th^ gene and *g_i_*(**t**) = (*g_i_*(*t_1_*), … , *g_i_*(*t_T_*))^Τ^. Note the difference between equations (50) and (1). In equation (1), the regulatees can themselves act as regulators, corresponding to genes coding for transcription factors acting on other genes. In equation (50), regulators (**Z**) and regulatees $\left(\dot{\text{x}}\right)$ are separated in what is effectively a bi-partite regulatory network structure. The ODE in equation (49) depends on the state variables **x***_i_* only by a linear decay term *δ_i_*. Consider a differencing matrix **D**, where

(51) $$\text{D}=ϒ\left[\begin{array}{cccccc} -1 & 1 & 0 & \ldots & \ldots & 0\\ -1 & 0 & 1 & 0 & \ldots & 0\\ 0 & -1 & \ddots & 1 & \ddots & \vdots \\ \vdots & \ddots & \ddots & \ddots & \ddots & \vdots \\ \vdots & \ddots & \ddots & \ddots & \ddots & \vdots \\ 0 & \ldots & \ldots & \ldots & -1 & 1 \end{array}\right],$$

and $ϒ=\mathrm{diag}\left(\frac{1}{t_{2}-t_{1}},\frac{1}{t_{3}-t_{1}},\frac{1}{t_{4}-t_{2}},\ldots ,\frac{1}{t_{T}-t_{T-2}},\frac{1}{t_{T}-t_{T-1}}\right)$. We can then approximate equation (49) as

(52) $$\text{D}{\text{x}}_{i}=g_{i}\left(\text{Z},\rho_{i},\text{t}\right)-\delta {\text{x}}_{i}.$$

To demonstrate how **Dx***_i_* is computed, as an example let us consider **x***_i_* = (*x*(*t*_1_), … , *x*(*t*_5_))^Τ^ and **t** = (1, 2, … , 5)^Τ^. Then,

(53) $$\begin{array}{l} \text{D}{\text{x}}_{i}=\left[\begin{array}{ccccc} \frac{1}{2-1} & & & & \\ & \frac{1}{3-1} & & & \\ & & \frac{1}{4-2} & & \\ & & & \frac{1}{5-3} & \\ & & & & \frac{1}{5-4} \end{array}\right]\times \left[\begin{array}{ccccc} -1 & 1 & 0 & 0 & 0\\ -1 & 0 & 1 & 0 & 0\\ 0 & -1 & 0 & 1 & 0\\ 0 & 0 & -1 & 0 & 1\\ 0 & 0 & 0 & -1 & 1 \end{array}\right]\;\left[\begin{array}{c} x\left(1\right)\\ x\left(2\right)\\ x\left(3\right)\\ x\left(4\right)\\ x\left(5\right) \end{array}\right]\\ =\left[\frac{-x\left(1\right)+x\left(2\right)}{1},\;\frac{-x\left(1\right)+x\left(3\right)}{2},\;\frac{-x\left(2\right)+x\left(4\right)}{2},\right.{\left.\frac{-x\left(3\right)+x\left(5\right)}{2},\frac{-x\left(4\right)+x\left(5\right)}{1}\right]}^{T}. \end{array}$$

Writing **P** = **D **+ *δ_i_* **I** (**I** here is the identity matrix) gives us the following penalty to be incorporated into the likelihood term

(54) $$\Omega \left({\text{x}}_{i}\right)=||\text{P}{\text{x}}_{i}-g_{i}\left(\text{Z},\rho_{i},\text{t}\right)|{|}^{2}.$$

Equation (52) implies that **Px***_i_* − *g_i_*(**Z**, ***ρ****_i_*, **t**) = 0. Rather than solving this equation explicitly, it is used as a penalty term within a regression context, i.e., the $||f|{|}_{H}^{2}$ term in equation (43) will be replaced by equation (54). However, equation (54) cannot be expressed as a norm of **x***_i_* within the RKHS framework, since **x***_i_* = **0** does not necessarily imply that **Ω**(**x***_i_*) = 0. The authors therefore transform the state variables **x***_i_* (and subsequently **y***_i_*) in order to make them compatible. Consider instead

(55) $${\tilde{\text{x}}}_{i}={\text{x}}_{i}-{\text{P}}^{-1}g_{i}\left(\text{Z},\rho_{i},\text{t}\right).$$

It is straightforward to see that multiplying both sides of equation (55) by **P** and taking squared norms gives us the exact form of equation (54) $\left({∥\text{P}{\tilde{\text{x}}}_{i}∥}^{2}={∥\text{P}{\text{x}}_{i}-g_{i}\left(\text{Z},\rho_{i},\text{t}\right)∥}^{2}\right)$. Likewise, the data are transformed by

(56) $${\tilde{\text{y}}}_{i}={\text{y}}_{i}-{\text{P}}^{-1}g_{i}\left(\text{Z},\rho_{i},\text{t}\right),$$

to correspond with ${\tilde{\text{x}}}_{i}$. The penalty term in equation (54) now becomes

(57) $$\Omega \left({\tilde{\text{x}}}_{i}\right)=||\text{P}{\tilde{\text{x}}}_{i}|{|}^{2}=\langle \text{P}{\tilde{\text{x}}}_{i},\text{P}{\tilde{\text{x}}}_{i}\rangle ={\tilde{\text{x}}}_{i}^{T}{\text{P}}^{T}\text{P}{\tilde{\text{x}}}_{i}.$$

Equation (57) is now a proper norm, since when ${\tilde{\text{x}}}_{i}=\text{0}$, this implies $\Omega \left({\tilde{\text{x}}}_{i}\right)=0$. Denote **K** = (**P**^Τ^**P**)^−1^. **K** is a matrix of kernel elements that define a unique RKHS. Hence,

(58) $$\Omega \left({\tilde{\text{x}}}_{i}\right)=||{\tilde{\text{x}}}_{i}|{|}_{H}^{2}={\text{c}}^{T}\text{Kc},$$

[where **c** is given in equation (47), and equation 58 is used as the term in the far right of equation 46, see Section 2.6 for details]. By using equations (47) and (48), we obtain closed-form expressions for the transformed state variables [and the original expressions can be recovered using equation (55)]

(59) $${\tilde{\text{x}}}_{i}=\text{K}{\left(\text{K}+2\lambda_{i}\Sigma \right)}^{-1}{\text{y}}_{i},$$

where *λ* is a penalty parameter, and **Σ** is the covariance matrix of the data [which generalizes equation (47) since the observational error of our data may not be independent between species]. In practice, not all ODEs are of the form in equation (50), which only depends on the state variables by a linear decay term. Hence, the authors need to transform any ODE that is not of this form into 2 parts. Terms in part (1) will have a dependency on the state variables only by a linear decay term and can be modeled using the RKHS method and estimated by equation (59). Terms in part (2) cannot fit this framework and are modeled using splines. For example, consider $\left[\dot{V}\right]$ in the FitzHugh–Nagumo ODEs (for more details, see Section 4)

(60) $$\left[\dot{V}\right]=\psi \left(\left[V\right]-\frac{{\left[V\right]}^{3}}{3}+\left[R\right]\right),$$

where the square brackets denote the time-dependent concentration for that species, the dot over the V is shorthand for the temporal derivative $\frac{d}{\mathrm{dt}}$ of V and *ψ* is a parameter. Since the state variables do not only depend on a linear decay term, equation (60) needs to be transformed. Part (1) will be expressed by $\left[\dot{V}\right]-\psi \left[V\right]$, where now the dependency on the state variables is only by a linear decay term and hence can be fitted using the RKHS method. Part (2) will be $\psi \left(-\frac{{\left[\hat{V}\right]}^{3}}{3}+\left[\hat{R}\right]\right)$, which is fitted using splines, where $\left[\hat{V}\right]$ and $\left[\hat{R}\right]$ are spline estimates for [*V*] and [*R*], respectively.

The penalized log-likelihood function can now be expressed by

(61) $$l\left(\rho_{i},\delta_{i},\Sigma ,\alpha_{i},\text{c}|{\tilde{\text{y}}}_{i}\right)=\overset{n}{\underset{i=1}{\sum }}\left[-\frac{1}{2}{\left({\tilde{\text{y}}}_{i}-{\tilde{\text{x}}}_{i}\right)}^{T}\Sigma^{-1}\left({\tilde{\text{y}}}_{i}-{\tilde{\text{x}}}_{i}\right)-\frac{1}{2}\ln|\Sigma |\right]-\overset{n}{\underset{i=1}{\sum }}\lambda_{i}\Omega \left({\tilde{\text{x}}}_{i}\right),$$

where ***α**_i_* is the vector containing the coefficients from the spline interpolant for species *i*, see equation (26). Given that the gradient matching is dependent on the differencing operator, it is important to note that points further apart in time will produce continually poorer estimates of the gradient and thus poorer gradient matching. González et al. (2013) attempt to circumvent this issue by data augmentation. They infer the latent variables at additional unobserved timepoints with the expectation maximization (EM) algorithm, which emulates more datapoints, in order to obtain more accurate gradient estimates. Parameter estimation in an approximate penalized maximum likelihood sense can be carried out with standard non-linear optimization algorithms, such as quasi-Newton or conjugate gradients.

## 3 Summary of Methods

This section provides a brief summary of the methods throughout the review, as described in Section 2. Since many methods and settings are used in this review for comparison purposes, for ease of reading, abbreviations are used. Table 3 is a reference for those methods and an overview of the methods follows.

INF (Section 2.1): this method conducts parameter inference using adaptive gradient matching and Gaussian processes. The penalty mismatch parameter ***γ*** (where ***γ*** is the vector of mismatch penalty parameter values at different “temperatures”) is inferred rather than tempered.
LB2 (Sections 2.1 and 2.2): this method conducts parameter inference using adaptive gradient matching and Gaussian processes. The penalty mismatch parameter ***γ*** is tempered in log base 2 increments, see Table 1 for details.
LB10 (Sections 2.1 and 2.2): as with LB2, parameter inference is conducted using adaptive gradient matching and Gaussian processes; however, the penalty mismatch parameter ***γ*** is tempered in log base 10 increments, see Table 1 for details.
C&S (Section 2.4): parameter inference is carried out using adaptive gradient matching and tempering of the mismatch parameter. The choice of interpolation scheme is B-splines.
RAM (Section 2.5): this technique uses a non-Bayesian optimization process for parameter inference. The method penalizes the difference between the gradients using splines and a hierarchical 3 level regularization approach is used to configure the tuning parameters.
GON (Section 2.7): parameter inference is conducted in a non-Bayesian fashion, implementing a reproducing kernel Hilbert space (RKHS) and penalized likelihood approach. Comparisons between RKHS and GPs have been previously explored conceptually [for example, see Rasmussen and Williams (2006) and Murphy (2012)], and in this review we analyze them empirically in the specific context of gradient matching. The RKHS gradient matching method in González et al. (2013) obtains the interpolant gradient using a differencing operator.

**Table 3:** **Abbreviations of the methods used throughout this review**.

Abbreviation	Method	Reference
GON	Reproducing kernel Hilbert space and penalized likelihood	González et al. (2013)
RAM	Splines and hierarchical regularization	Ramsay et al. (2007)
INF	Inference of the gradient mismatch parameter using GPs	Dondelinger et al. (2013)
LB2	Tempered mismatch parameter using GPs in log base 2 increments	Macdonald and Husmeier (2015)
LB10	Tempered mismatch parameter using GPs in log base 10 increments	Macdonald and Husmeier (2015)
C&S	Tempered mismatch parameter using splines- based smooth functional tempering (SFT)	Campbell and Steele (2012)

Table 4 outlines particular settings with some of the methods in Table 3. The ranges of the penalty parameter for *γ*, for LB2 and LB10 methods, are given in Table 1. The increments are equidistant on the log scale. The M *β_i_*s from 0 to 1 are set by taking a series of equidistant M values and raising them to the power 5 [Friel and Pettitt (2008)].

**Table 4:** **Particular settings of Campbell and Steele (2012)’s method**.

Abbreviation	Definition	Details
10C	10 Chains	When comparing methods, it was of interest to see how the performance depended on the number of parallel MCMC chains, as originally the authors used 4 chains
Obs20	20 Observations	Originally, the authors used 401 observations. This was reduced to a dataset size more usual with these types of experiments to observe the dependency of the methods on the amount of data
15K	15 Knots	The C&S method uses B-splines interpolation. The original tuning parameters from the authors’ paper were changed to observe the sensitivity of the parameter estimation from these tuning parameters
P3	Polynomial order 3 (Cubic Spline)	The original polynomial order is 5. Again, this was changed to observe the sensitivity of the parameter estimation from these tuning parameters

## 4 Data

### 4.1 FitzHugh–Nagumo

These equations model the voltage potential across the cell membrane of the axon of giant squid neurons [FitzHugh (1961); Nagumo et al. (1962)]. There are two “species”: voltage (V) and recovery variable (R), and 3 parameters; *α*, *β*, and *ψ*. The square brackets denote the time-dependent concentration for that species and a dot over a symbol is shorthand for the temporal derivative $\frac{d}{\mathrm{dt}}$ of that symbol:

(62) $$\begin{array}{l} \left[\dot{V}\right]=\psi \left(\left[V\right]-\frac{{\left[V\right]}^{3}}{3}+\left[R\right]\right);\;\left[\dot{R}\right]=-\frac{1}{\psi }\left(\left[V\right]-\alpha +\beta *\left[R\right]\right). \end{array}$$

**Figure 4:**
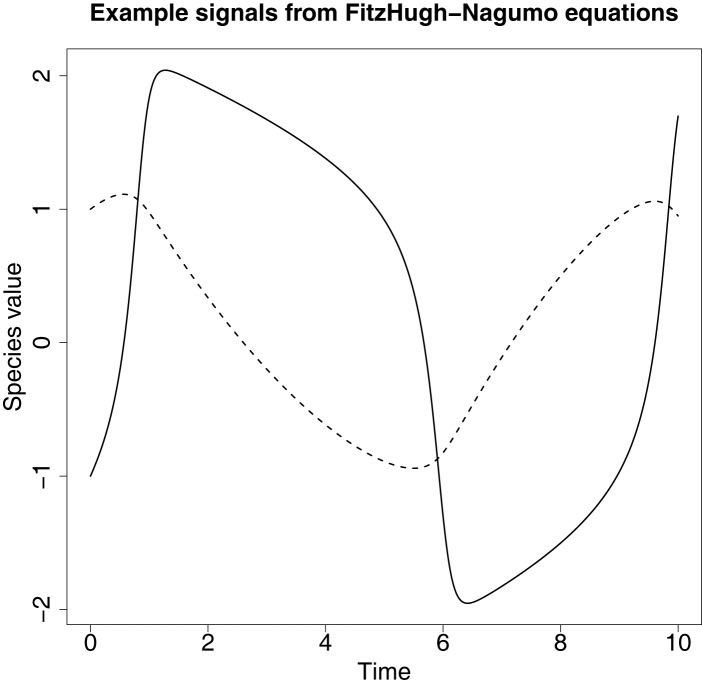
**An example of the signals produced from the FitzHugh–Nagumo ODEs in equation (62)**. The solid line represents the signal for species V and the dashed line represents the signal for species R.

An example of the signals produced from these ODEs can be found in Figure 4.

### 4.2 Protein Signaling Transduction Pathway

**Figure 5:**
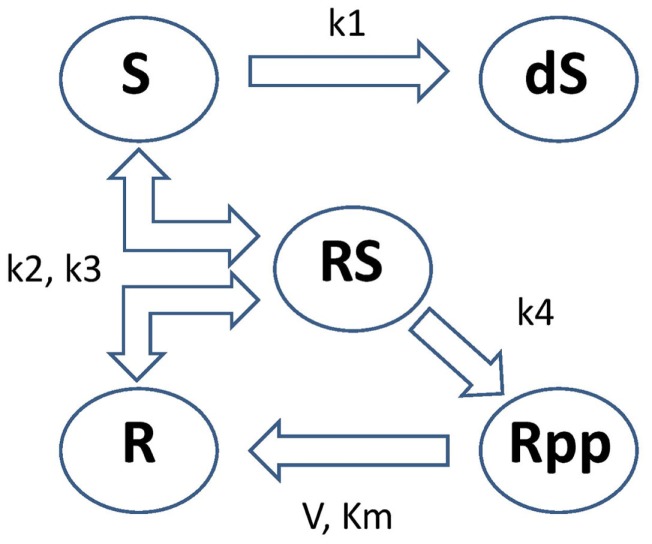
**Graphical representation of the protein signaling transduction pathway in equations (63)–(67)**. There are 5 “species” (*S, dS, R, RS, Rpp*) and 6 parameters (*k*_1_, *k*_2_, *k*_3_, *k*_4_, *V*, *K_m_*). The system describes the phosphorylation of a protein, *R* → *Rpp* [equation (67)], catalyzed by an enzyme *S*, via an active protein complex [*RS*, equation (66)], where the enzyme is subject to degradation [S → *dS*, equation (64)]. Figure adapted from Vyshemirsky and Girolami (2008).

These equations model protein signaling transduction pathways in a signal transduction cascade, where the free parameters are kinetic parameters governing how quickly the proteins (“species”) convert to one another [Vyshemirsky and Girolami (2008)]. There are 5 “species” (*S*, *dS*, *R*, *RS*, *Rpp*) and 6 parameters (*k*_1_, *k*_2_, *k*_3_, *k*_4_, *V*, *K_m_*). The system describes the phosphorylation of a protein, *R * → *Rpp* [equation (67)], catalyzed by an enzyme *S*, via an active protein complex [*RS*, equation (66)], where the enzyme is subject to degradation [*S* → *dS*, equation (64)]. The chemical kinetics are described by a combination of mass action kinetics [equations (63), (64), and (66)] and Michaelis–Menten kinetics [equations (65) and (67)]. A graphical representation of this system can be seen in Figure 5. The square brackets denote the time-dependent concentration for that species and a dot over a symbol is shorthand for the temporal derivative $\frac{d}{\mathrm{dt}}$ of that symbol:

(63) $$\left[\dot{S}\right]=-k_{1}*\left[S\right]-k_{2}*\left[S\right]*\left[R\right]+k_{3}*\left[\mathrm{RS}\right],$$

(64) $$\left[\dot{d}s\right]=k_{1}*\left[S\right],$$

(65) $$\left[\dot{R}\right]=-k_{2}*\left[S\right]*\left[R\right]+k_{3}*\left[\mathrm{RS}\right]+\frac{V*\left[\mathrm{Rpp}\right]}{K_{m}+\left[\mathrm{Rpp}\right]},$$

(66) $$\left[\dot{R}S\right]=k_{2}*\left[S\right]*\left[R\right]-k_{3}*\left[\mathrm{RS}\right]-k_{4}*\left[\mathrm{RS}\right],$$

(67) $$\left[R\dot{p}p\right]=k_{4}*\left[\mathrm{RS}\right]-\frac{V*\left[\mathrm{Rpp}\right]}{K_{m}+\left[\mathrm{Rpp}\right]}.$$

**Figure 6:**
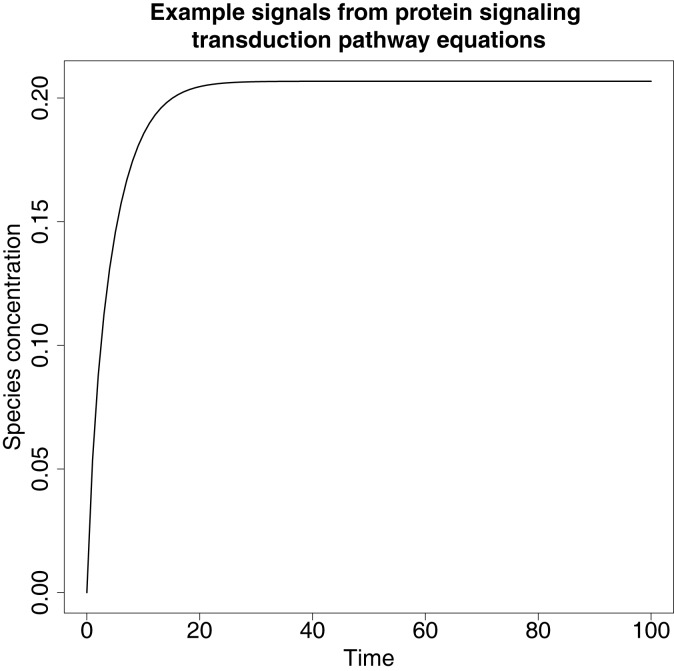
**An example of a signal produced from the protein signaling transduction pathway in equation (64)**. The signal represents species dS and shows a rapid change in concentration before it plateaus, which is a feature typical of the remaining species’ signals in equations (63)–(67).

An example of a typical signal produced from these ODEs can be found in Figure 6.

## 5 Simulation

For those methods for which software was unavailable at the time [Ramsay et al. (2007); González et al. (2013)], results were compared directly with the results from the original publications. To this end, test data were generated in the same way as described by the authors. For methods for which software was available at the time [Campbell and Steele (2012); Dondelinger et al. (2013); Macdonald and Husmeier (2015)], the evaluation was repeated twice, first on data equivalent to those used in the original publications, and again on new data generated with different (more realistic) parameter settings. For comparisons with Bayesian methods, the authors’ specifications for the priors on the ODE parameters were used. For comparisons with non-Bayesian methods, the methods of Dondelinger et al. (2013) and Macdonald and Husmeier (2015) were applied with the parameter prior from Campbell and Steele (2012), since the ODE model was the same.

### 5.1 Reproducing Kernel Hilbert Space Method (Section 2.7)

The method was tested on the FitzHugh–Nagumo data (see Section 4) with the following parameters: *α* = 0.2; *β* = 0.2, and *ψ * = 3. Starting from initial values of (−1, −1) for the two “species”, 50 timepoints were generated over the time course [0, 20], producing 2 periods, with iid Gaussian noise (SD = 0.1) added. Fifty independent datasets were generated in this way.

### 5.2 Splines and Hierarchical Regularization Method (Section 2.5)

This method was included in the study by González et al. (2013), and the results in this review are from the original paper. For a proper comparison, the methods of Dondelinger et al. (2013) and Macdonald and Husmeier (2015) were applied in the same way as in for the comparison with González et al. (2013).

### 5.3 Tempered Mismatch Parameter Using Splines-Based Smooth Functional Tempering (Section 2.4)

The method was tested on the FitzHugh–Nagumo system with the following parameter settings: *α* = 0.2; *β* = 0.2, and *ψ* = 3, starting from initial values of (−1, 1) for the two “species” [note the different starting values to the set-up in González et al. (2013)]. Four hundred and one observations were simulated over the time course [0, 20] (producing 2 periods) and Gaussian noise was added with SD {0.5, 0.4} to each respective “species”. The original settings were used for inferring the ODE parameters: splines of polynomial order 5 with 301 knots; four parallel tempering chains associated with gradient mismatch parameters {10, 100, 1000, 10,000}; parameter prior distributions for the ODE parameters: *α* ∼ *N*(0, 0.4^2^), β ∼ *N*(0, 0.4^2^), and $\psi ∼\chi_{2}^{2}$.

In addition to comparing the methods of Dondelinger et al. (2013) and Macdonald and Husmeier (2015) with these original settings, the following modifications were made to test the robustness of the procedures with respect to these (rather arbitrary) choices. The number of observations was reduced from 401 to 20 over the time course [0, 10] (producing 1 period) to reflect more closely the amount of data typically available from current systems biology projects. For these smaller datasets, the number of knots for the splines was reduced to 15 (keeping the same proportionality of knots to datapoints as before), and a different polynomial order was tested: 3 instead of 5. Due to the high computational costs of the Campbell and Steele (2012) method (roughly $1\frac{1}{2}$ weeks for a run), only 3 MCMC simulations on 3 independent datasets could be run. The respective posterior samples were combined, to approximately marginalize over datasets, and thereby remove their potential particularities. For a fair comparison, the tempering schedule in Campbell and Steele (2012) was applied to the methods of Dondelinger et al. (2013) and Macdonald and Husmeier (2015) such that 4 parallel chains were used rather than 10.

### 5.4 Inference of the Gradient Mismatch Parameter Using GPs (Section 2.1)

The methods of Dondelinger et al. (2013) and Macdonald and Husmeier (2015) were applied in the same way as in the original publication of Dondelinger et al. (2013), selecting the same kernels and parameter/hyperparameter priors. Data were generated from the protein signal transduction pathway, described in Section 4, with the following settings; ODE parameters: (*k*_1_ = 0.07, *k*_2_ = 0.6, *k*_3_ = 0.05, *k*_4_ = 0.3, *V*  = 0.017, *K_m_* = 0.3); initial values of the species: (*S * = 1, *dS * = 0, *R * = 1, *RS * = 0, *Rpp * = 0); 15 timepoints covering one period, {0, 1, 2, 4, 5, 7, 10, 15, 20, 30, 40, 50, 60, 80, 100}. Multiplicative iid Gaussian noise of SD = 0.1 was used to distort the signals, in order to reflect observational error that would be obtained in experiments. For Bayesian inference, a Γ(4, 0.5) prior was used for the ODE parameters. For the GP, we used the same kernel as in Dondelinger et al. (2013); see below for details. In addition to this ODE system, these methods were also applied to the set-ups previously described for the FitzHugh–Nagumo model.

### 5.5 Choice of Kernel

For the GP, a suitable kernel needs to be chosen, which defines a prior distribution in function space. Two kernels are considered in this review [to match the authors’ set-ups in Dondelinger et al. (2013)], the radial basis function (RBF) kernel

(68) $$k\left(t_{i},t_{j}\right)=\sigma_{\mathrm{RBF}}^{2}\exp\left(-\frac{{\left(t_{i}-t_{j}\right)}^{2}}{2l^{2}}\right),$$

with hyperparameters $\sigma_{\mathrm{RBF}}^{2}$ and *l*^2^, and the sigmoid variance kernel

(69) $$k\left(t_{i},t_{j}\right)=\sigma_{\mathrm{sig}}^{2}\arcsin\frac{a+\left(bt_{i}t_{j}\right)}{\sqrt{\left(a+\left(bt_{i}t_{i}\right)+1\right)\left(a+\left(bt_{j}t_{j}\right)+1\right)}},$$

with hyperparameters $\sigma_{\mathrm{sig}}^{2}$, *a* and *b* [Rasmussen and Williams (2006)].

To choose initial values for the hyperparameters, a standard GP regression model (i.e., without the ODE part) is fitted using maximum likelihood. The interpolant is then inspected to decide whether it adequately represents the prior knowledge of the signal. For the data generated from the FitzHugh–Nagumo model, the RBF kernel provides a good fit to the data. For the protein signaling transduction pathway, the non-stationary nature of the data is not represented properly with the RBF kernel, which is stationary [Rasmussen and Williams (2006)], in confirmation of the findings in Dondelinger et al. (2013). Following Dondelinger et al. (2013), the sigmoid variance kernel was used, which is non-stationary [Rasmussen and Williams (2006)] and this provided a considerably improved fit to the data.

### 5.6 Other Settings

Finally, the values for the variance mismatch parameter of the gradients, *γ*, needs to be configured for the method in Macdonald and Husmeier (2015). Log base 2 and log base 10 increments were used (initializing at 1), since studies that indicate reasonable values are limited [see Calderhead et al. (2008) and Friel and Pettitt (2008)]. All parameters were initialized with a random draw from the respective priors (apart from GON and RAM, which did not use priors).

## 6 Results

We present the results in the same way the authors of the methods we are comparing presented them in the original papers. For the methods we had obtained the authors’ code for, we also present the root mean square (RMS) values in function space. First, the signal was reconstructed with the sampled parameters and then the true signal was subtracted (signal created with true parameters and no observational noise added). The RMS was calculated on these residuals. It is important to assess the methods on this criterion as well as looking at the parameter uncertainty, as some parameters might only be weakly identifiable, corresponding to ridges in the likelihood landscape. In other words, large uncertainty in parameter estimates may not necessarily imply a poor performance by a method, if the reconstructed signals for all groups of sampled parameters were close to the truth.

All distributions of the results in this section are displayed graphically as boxplots, which display whiskers that extend from the lower (*Q*1) and upper (*Q*3) quartiles of the box, to boundaries defined by *Q*1 − 1.5(*Q*3 − *Q*1) and *Q*3 + 1.5(*Q*3 − *Q*1). All values outside these boundaries are considered outliers and drawn as a circle.

### 6.1 Reproducing Kernel Hilbert Space (Section 2.7) and Hierarchical Regularization (Section 2.5) Methods

**Figure 7:**
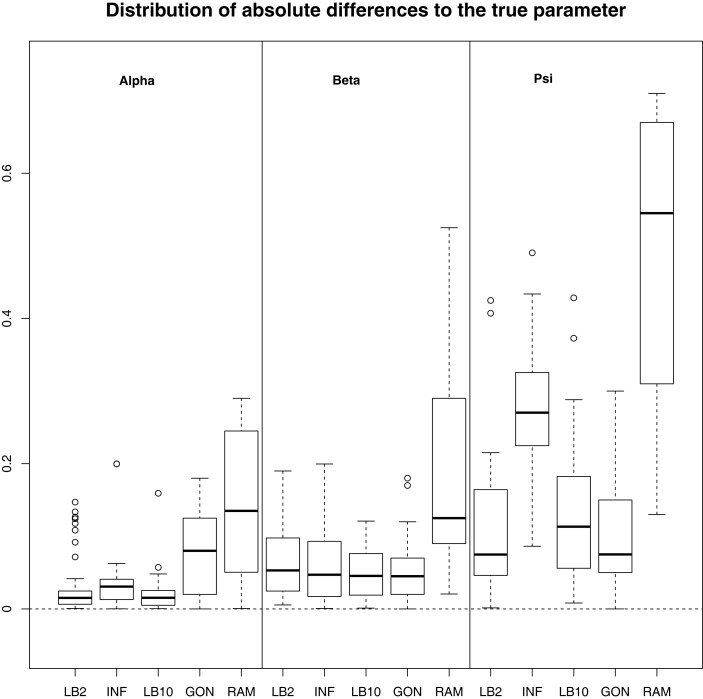
**Boxplots of the distributions of the absolute differences of an estimate to the true parameter over 50 datasets**. The three sections from left to right represent the parameters α, β, and ψ from equation (62). Within each section, the boxplots from left to right are: LB2 method, INF method, LB10 method, GON’s method [boxplot reconstructed from González et al. (2013)], and RAM’s method [boxplot reconstructed from González et al. (2013)]. For an explanation of the boxplot form, see the beginning of Section 6. Figure reconstructed from Macdonald and Husmeier (2015).

For this configuration, to judge the performance of the methods, we used the same concept as in GON to examine our results. For each parameter, the absolute value of the difference between an estimate and the true parameter $\left(\left|{\hat{\theta }}_{i}-\theta_{i}\right|\right)$ was computed and the distribution across the datasets was examined. For the LB2, LB10, and INF methods, the median of the sampled parameters was used since it is a robust estimator. Looking at Figure 7, the LB2, LB10, and INF methods do as well as the GON method for 2 parameters (INF doing slightly worse for *ψ*) and outperform it for 1 parameter. All methods outperform the RAM method.

### 6.2 Tempered Mismatch Parameter Using Splines-Based Smooth Functional Tempering (Section 2.4)

**Figure 8:**
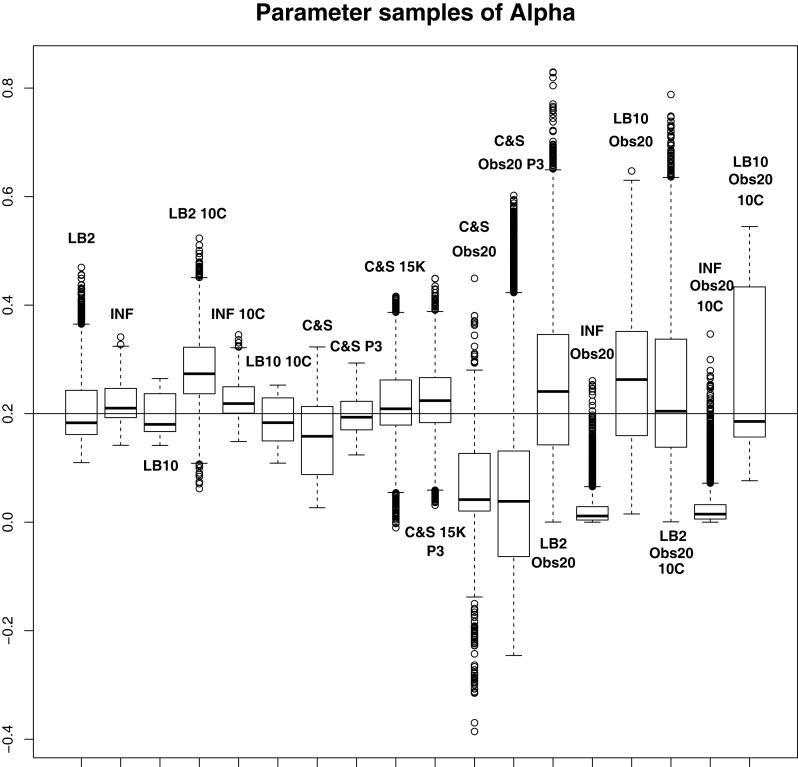
**Average posterior distributions of parameter **α** from equation (62) over 3 datasets**. From left to right: LB2, INF, LB10, LB2 10C, INF 10C, LB10 10C, C&S, C&S P3, C&S 15K, C&S 15K P3, C&S Obs20, C&S Obs20 P3, LB2 Obs20, INF Obs20, LB10 Obs20, LB2 Obs20 10C, INF Obs20 10C, and LB10 Obs20 10C. The solid line is the true parameter. For definitions, see Tables 3 and 4. For an explanation of the boxplot form, see the beginning of Section 6. Figure reconstructed from Macdonald and Husmeier (2015).

**Figure 9:**
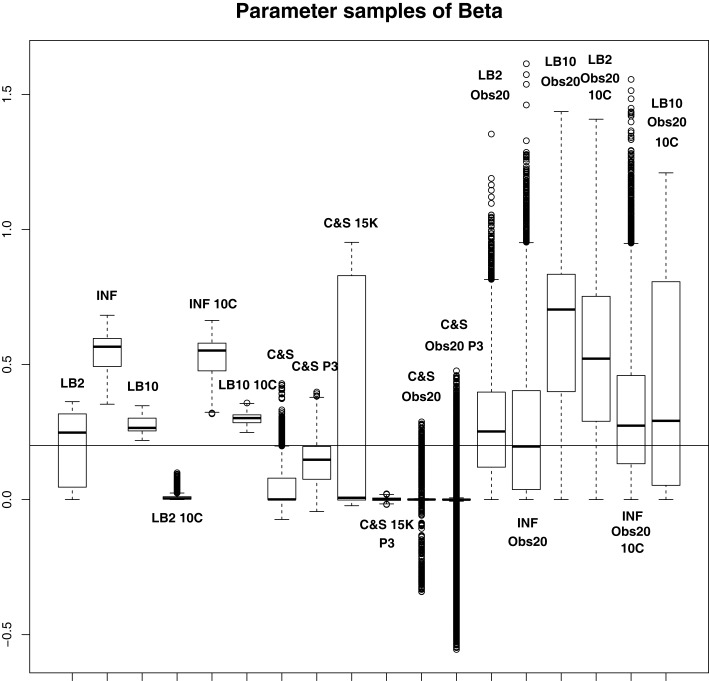
**Average posterior distributions of parameter **β** from equation (62) over 3 datasets**. From left to right: LB2, INF, LB10, LB2 10C, INF 10C, LB10 10C, C&S, C&S P3, C&S 15K, C&S 15K P3, C&S Obs20, C&S Obs20 P3, LB2 Obs20, INF Obs20, LB10 Obs20, LB2 Obs20 10C, INF Obs20 10C, and LB10 Obs20 10C. The solid line is the true parameter. For definitions, see Tables 3 and 4. For an explanation of the boxplot form, see the beginning of Section 6. Figure reconstructed from Macdonald and Husmeier (2015).

**Figure 10:**
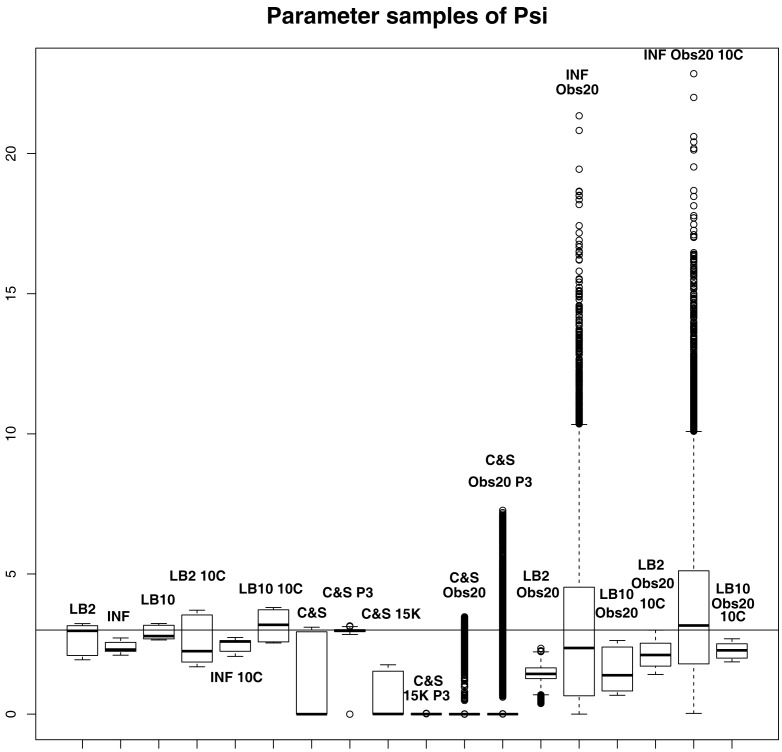
**Average posterior distributions of parameter **ψ** from equation (62) over 3 datasets**. From left to right: LB2, INF, LB10, LB2 10C, INF 10C, LB10 10C, C&S, C&S P3, C&S 15K, C&S 15K P3, C&S Obs20, C&S Obs20 P3, LB2 Obs20, INF Obs20, LB10 Obs20, LB2 Obs20 10C, INF Obs20 10C, and LB10 Obs20 10C. The solid line is the true parameter. For definitions, see Tables 3 and 4. For an explanation of the boxplot form, see the beginning of Section 6. Figure reconstructed from Macdonald and Husmeier (2015).

For this set-up, the entire posterior distributions were examined. The posterior distributions were averaged over datasets in order to present the overall performance of each method, not confounded by the particular observational error that was added to a dataset. The C&S method shows good performance over all parameters in the one case where the number of observations is 401, the number of knots is 301, and the polynomial order is 3 (cubic spline), since the bulk of the average posterior distributions of the sampled parameters surrounds the true parameters in Figures 8 and 10 and is close to the true parameter in Figure 9. However, these settings require a great deal of “hand-tuning” or time expensive cross-validation and would be very difficult to set when using real data. The sensitivity of the splines-based method can be seen in the other settings, where the results deteriorate. It is also important to note that when the dataset size was reduced, the cubic spline performed very badly. This inconsistency makes these methods very difficult to apply in practice. The LB2, LB10, and INF methods consistently outperform the C&S method with the bulk of the average posterior distributions overlapping or being closer to the true parameters. On the set-up with 20 observations, for 4 chains and 10 chains, the INF method produced largely different estimates over the datasets, as depicted by the wide boxplots and long tails. The long tails in all of these distributions are due to the combination of estimates from different datasets.

**Figure 11:**
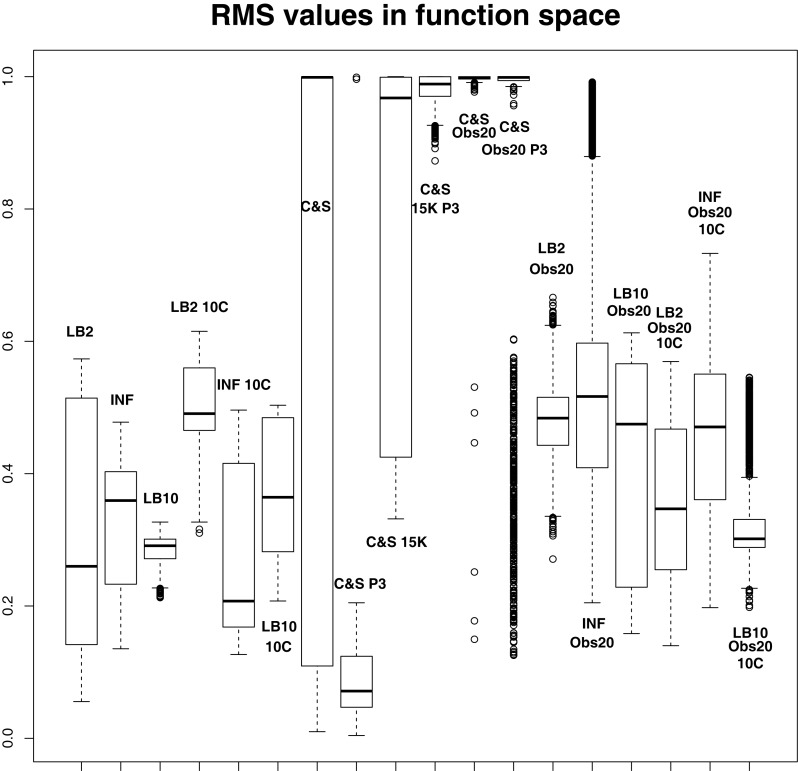
**Average posterior distributions of RMS values in function space, calculated on the residuals of the true signal (signal produced with true parameters and no observational error) minus the signal produced with the posterior sampled parameters**. For viewing purposes, a squashing function has been applied to the RMS values, see equation (70). Larger values show poorer reconstructed signals. From left to right: LB2, INF, LB10, LB2 10C, INF 10C, LB10 10C, C&S, C&S P3, C&S 15K, C&S 15K P3, C&S Obs20, C&S Obs20 P3, LB2 Obs20, INF Obs20, LB10 Obs20, LB2 Obs20 10C, INF Obs20 10C, and LB10 Obs20 10C. For definitions, see Tables 3 and 4. For an explanation of the boxplot form, see the beginning of Section 6.

By examining Figure 11, we can see how the methods perform in function space. The RMS values for some of the C&S set-ups were very large, so for graphical viewing purposes, we applied a squashing function

(70) $$f\left(\mathrm{RMS}\right)=\frac{\mathrm{RMS}}{1+\mathrm{RMS}},$$

where *f* (*RMS*) = *RMS* for *RMS* << 1, and *f* (*RMS*) = 1 for *RMS* → ∞. The RMS values have been monotonically transformed and values closer to 0 show better performance, whereas values closer to 1 show poorer performance. These results reinforce what we saw from the parameter estimates. The C&S performs well only in the one case where there was a large number of datapoints (401) and a cubic spline was used. The other set-ups for C&S perform very poorly, including the case where the cubic spline was used with a smaller dataset size. The LB2, INF, and LB10 methods perform well and similarly across the different set-ups, with LB10 performing slightly better in some scenarios.

### 6.3 Inference of the Gradient Mismatch Parameter Using GPs (Section 2.1)

**Figure 12:**
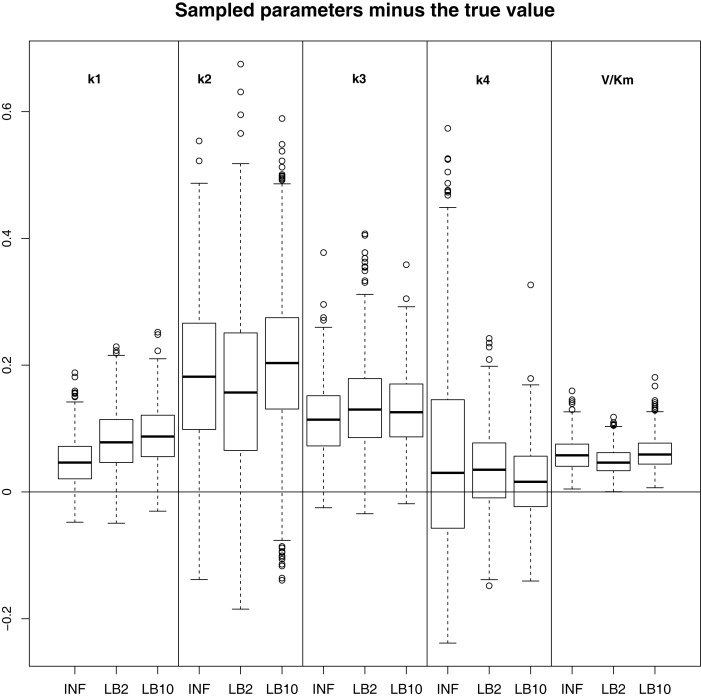
**Results from the dataset that showed the average RMS of the posterior parameter samples minus the true values for the INF, LB2, and LB10 methods**. The posterior distributions are of the sampled parameters from equations (63)–(67) minus their true values. The horizontal line shows zero difference. For an explanation of the boxplot form, see the beginning of Section 6. Figure reconstructed from Macdonald and Husmeier (2015).

In order to see how the tempering method in Macdonald and Husmeier (2015) performs in comparison to the INF method, we can examine the results from the protein signaling transduction pathway (see Section 4), as well as comparing the results in the previous set-ups. The distributions of the posterior parameter samples minus the true values for the protein signaling transduction pathway are shown in Figure 12. The INF method was unable to converge properly for some of the datasets. In order to present the average performance of the methods, for INF, LB2, and LB10, the root mean square (RMS) of the difference between the posterior parameter samples and the true values was calculated across all datasets. The results from the dataset which produced the median RMS are shown for each method.

**Figure 13:**
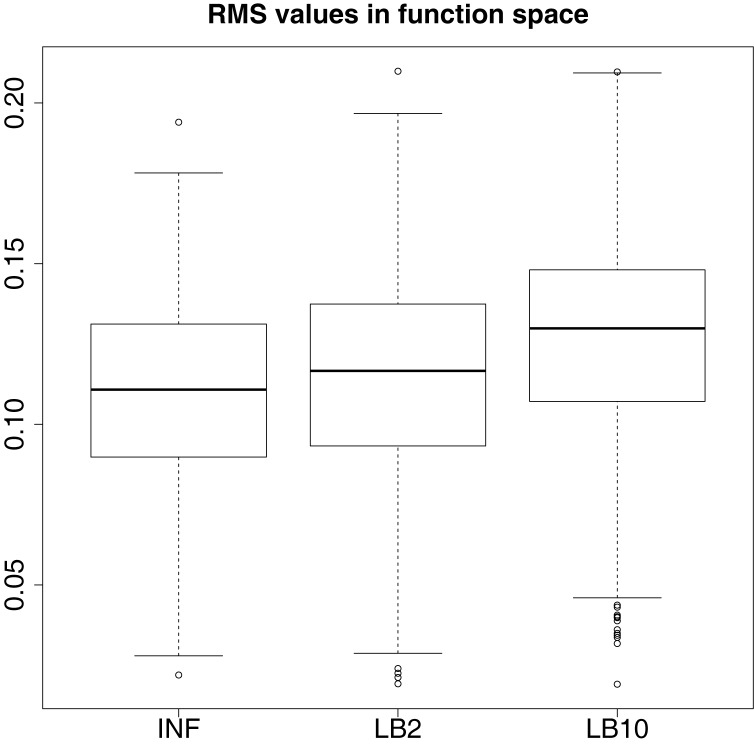
**Posterior distributions of RMS values for the ODE model in equations (63)–(67)**. The RMS values are calculated on the residuals of the true signal (signal produced with true parameters and no observational error) minus the signal produced from the sampled parameters. For an explanation of the boxplot form, see the beginning of Section 6.

By examining Figure 12, we can see that for each parameter, the bulk of the distributions is close to the true value and so the methods are performing reasonably. Overall, there does not appear to be a significant difference between the INF, LB2, and LB10 methods for this model. Figure 13 shows the distribution of RMS values for INF, LB2, and LB10 methods in terms of deviance from the true time series. All three methods perform similarly to one another, with RMS values close to zero.

**Figure 14:**
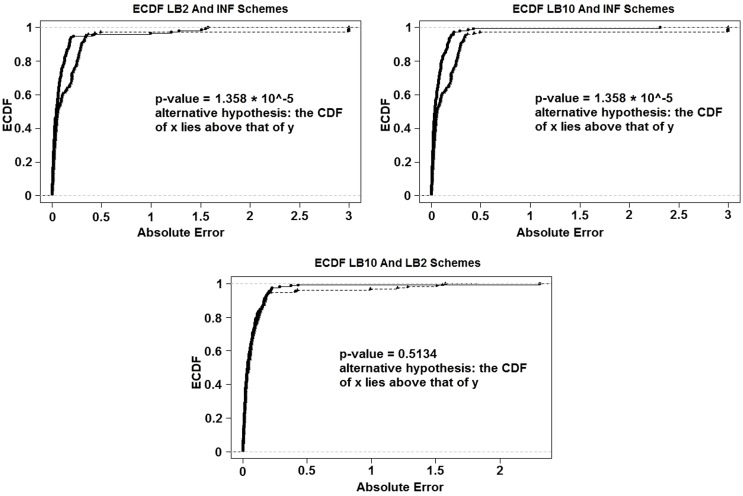
**ECDFs of the absolute errors of the parameter estimation**. Top left – ECDFs for LB2 and INF, top right – ECDFs for LB10 and INF, and bottom – ECDFs for LB10 and LB2. Included are the p-values for 2-sample, 1-sided Kolmogorov–Smirnov tests. For definitions, see Tables 3 and 4. Figure reconstructed from Macdonald and Husmeier (2015).

For the set-up in Sections 2.7 and 2.5: Figure 14 shows the Expected Cumulative Distribution Functions (ECDFs) of the absolute difference of the posterior parameter samples to the true values, for INF, LB2, and LB10. Included are the p-values for 2-sample, 1-sided Kolmogorov–Smirnov tests. If a distribution’s ECDF is significantly higher than another, this constitutes as better parameter estimation. A higher curve means that a method has more values that lie in the lower range of absolute error.

Figure 14 shows that both the LB2 and LB10 methods outperform the INF method, shown by p-values of less than the standard significance level of 0.05. Therefore, we conclude that the CDFs for LB2 and LB10 are significantly higher than those for INF. Since we are dealing with absolute errors, this means that the parameter estimates from the LB2 and LB10 methods are closer to the true parameters than the INF method. The LB2 and LB10 methods show no significant difference to each other.

For the set-up in Section 2.4: The LB2 and LB10 methods do well over all the parameters and dataset sizes, with most of the mass of the distributions surrounding or being situated close to the true parameters. The LB2 does better than the LB10 for 4 parallel chains (distributions overlapping the true parameter for all three parameters) and the LB10 outperforms the LB2 for 10 parallel chains (distribution overlapping true parameter in Figure 8, being closer to the true parameter in Figure 9, and narrower and more symmetric around the true parameter in Figure 10). The INF method’s bulk of parameter sample distributions is located close to the true parameters for all dataset sizes. However, the decrease in uncertainty is at the expense of bias. When reducing the dataset to 20 observations, for 4 and 10 chains, the inference deteriorates and is outperformed by the LB2 and LB10 methods. This could be due to the parallel tempering scheme constraining the mismatch between the gradients in chains closer to the posterior, allowing for better estimates of the parameters.

## 7 Discussion

We have carried out a comparative evaluation of various state-of-the-art gradient matching methods. These methods are based on different statistical modeling and inference paradigms: non-parametric Bayesian statistics with Gaussian processes (INF, LB2, and LB10), hierarchical regularization using splines interpolation (RAM), splines-based smooth functional tempering (C&S), and penalized likelihood based on reproducing kernel Hilbert spaces (GON). We have also compared the antagonistic paradigms of Bayesian inference (INF) versus parallel tempering (LB2 and LB10) of slack parameters in the specific context of adaptive gradient matching. We discuss aspects of the methodology and empirical findings separately.

### 7.1 Methodology

The GON method, due to the RKHS framework, is fast to implement. This is an attractive property, since the main motivation for gradient matching methods was to obtain a computational speed-up over techniques that calculate the numerical solution to the ODEs. This method hinges on obtaining better estimates of the interpolant gradient by use of the EM algorithm, so great care needs to be had when implementing this step. Care also needs to be exercised in fitting the splines estimates of the species for terms of the ODEs that cannot be fit with the RKHS approach. This step is not optimized within the penalized likelihood of equation (61) and so poor splines estimates could deteriorate the results. The 3 level hierarchy of the RAM method, for first configuring the tuning parameters and then for performing parameter estimation, is sensible. Since gradient matching methods rely on a good estimate of the interpolant, focusing on the tuning parameters should achieve more robust parameter estimates of the ODEs. This 3 level approach, however, does increase the computational complexity and the RAM method does not achieve a good speed-up over the numerical solution methods. The C&S method’s use of parallel tempering of both the likelihood and gradient mismatch parameter is intuitive, and allows the MCMC to explore with a reduced chance of getting stuck on local optima. This method uses B-splines interpolation, which can be difficult in practice to configure the tuning parameters for. The INF method has the advantage that the interpolant hyperparameters can be inferred from the data, since it uses Gaussian process interpolation. The inference approach to the gradient mismatch parameter, as opposed to tempering, however, could be overflexible and drive the parameter to values where the coupling between the gradients is too weak. The LB2 and LB10 methods avoid this by using a tempering scheme to drive the parameter to the theoretically correct value (0 corresponding to no mismatch between the gradients). However, currently, there is no way of optimizing the specific step size and increment schedule.

### 7.2 Empirical Findings

The GON method produces estimates that are close to the true parameters in terms of absolute uncertainty. This, however, was for the case with small observational noise (Gaussian iid noise SD = 0.1), and it would be interesting to see how the parameter estimation accuracy is affected by the increase of noise. The RAM method performs worse than the rest of the methods it was compared to, across all parameters. The C&S method does well only in the one case, where the number of observations is very high (higher than what would be expected in these types of experiments) and the tuning parameters are finely adjusted (which in practice is very difficult and time-consuming). When the number of observations was reduced, all settings for this method deteriorated significantly. It is important also to note that the settings that we found to be optimal were slightly different than in the original paper, which highlights the sensitivity and lack of robustness of the splines-based method. The INF method shows a reasonable performance in terms of consistently producing results close to the true parameters, across all the set-ups we have examined. However, this technique’s decrease in uncertainty is at the expense of bias. The LB2 and LB10 methods show the best performance across the set-ups. The parameter accuracy is unbiased across the different ODE models and the different settings of those models. The parallel tempering seems to be quite robust, performing similarly across the various set-ups. We have explored four different schedules for the parallel tempering scheme (as shown in Table 1). Overall, the performance of parallel tempering has been found to be reasonably robust with respect to a variation of the schedule.

## Conflict of Interest Statement

The authors declare that the research was conducted in the absence of any commercial or financial relationships that could be construed as a potential conflict of interest.

## Footnotes

^1^We do not make the baseline transcription rate explicit in our notation, but include it in the function *g*_i_(.).

^2^By “temperature”, we mean a tempering parameter that defines the degree of flattening of the likelihood. Formally, our “temperature” is equivalent to an inverse temperature in Statistical Physics.
